# NMR analysis suggests the terminal domains of Robo1 remain extended but are rigidified in the presence of heparan sulfate

**DOI:** 10.1038/s41598-022-18769-6

**Published:** 2022-08-30

**Authors:** Robert V. Williams, Chin Huang, Kelley W. Moremen, I. Jonathan Amster, James H. Prestegard

**Affiliations:** 1 Complex Carbohydrate Research Center and Department of Chemistry, University of Georgia, Athens, GA USA; 2 Complex Carbohydrate Research Center and Department of Biochemistry and Molecular Biology, University of Georgia, Athens, GA USA

**Keywords:** Carbohydrates, Biophysical chemistry

## Abstract

Human roundabout 1 (hRobo1) is an extracellular receptor glycoprotein that plays important roles in angiogenesis, organ development, and tumor progression. Interaction between hRobo1 and heparan sulfate (HS) has been shown to be essential for its biological activity. To better understand the effect of HS binding we engineered a lanthanide-binding peptide sequence (Loop) into the Ig2 domain of hRobo1. Native mass spectrometry was used to verify that loop introduction did not inhibit HS binding or conformational changes previously suggested by gas phase ion mobility measurements. NMR experiments measuring long-range pseudocontact shifts were then performed on ^13^C-methyl labeled hRobo1-Ig1-2-Loop in HS-bound and unbound forms. The magnitude of most PCSs for methyl groups in the Ig1 domain increase in the bound state confirming a change in the distribution of interdomain geometries. A grid search over Ig1 orientations to optimize the fit of data to a single conformer for both forms produced two similar structures, both of which differ from existing X-ray crystal structures and structures inferred from gas-phase ion mobility measurements. The structures and degree of fit suggest that the hRobo1-Ig1-2 structure changes slightly and becomes more rigid on HS binding. This may have implications for Robo-Slit signaling.

## Introduction

Human roundabout 1 (hRobo1) is a highly glycosylated extracellular receptor protein, first identified in Drosophila as important for immune system development^1^. Robo1 was later discovered to participate in angiogenesis and organ development^2,3^. hRobo1 achieves these biological functions through interaction with another secreted glycoprotein, Slit2, and heparan sulfate (HS). It has been suggested that a ternary signaling complex, involving the first two immunoglobulin-like (Ig1 and Ig2) domains of hRobo1, the second leucine-rich-repeat domain (D2) of Slit2 and HS, is key to its function^4^.

Much is known about the molecular basis for Robo1-Slit2 interactions. Early on, the binding interface between the two proteins was determined from a cocrystal structure of hRobo1-Ig1 and Slit2-D2^5^. More recently, studies of larger hRobo1 constructs have shed light on the mechanisms that propagate a signal produced on Slit2 binding to the inside of the cell. A combination of x-ray crystallography for hRobo1-Ig1-4 and small angle X-ray scattering (SAXS) of the entire hRobo1 ectodomain determined that full-length hRobo1 forms a tetrameric assembly which undergoes a large-scale conformation change upon binding Slit2^6^. Less is known about the structural effects of HS binding. Some information on the HS-binding site was revealed by an X-ray crystal structure of Drosophila Robo1-Ig1-2 in complex with a heparin-derived oligosaccharide of incompletely defined composition^1^. However, Slit2 was not in the crystal and the HS fragment bound between two molecules in the crystal structure, leaving details of the binding site in question. Hydroxy radical footprinting mass spectrometry further characterized the HS binding site, and solution nuclear magnetic resonance (NMR) spectroscopy provided a more detailed model of a complex between hRobo1-Ig1-2 and a synthetic HS tetrasaccharide, but a minimal perturbation of the protein structure was assumed^7,8^.

One intriguing result on the effect of HS binding on the Robo1 structure was reported from a study employing ion mobility mass spectrometry (IM-MS)^9^. hRobo1-Ig1-2 was observed to exist in two conformations, one significantly more compact than the available crystal structures, and one more consistent with elongated forms seen in the X-ray crystal structures. Analysis of hRobo1-Ig1-2 complexed with an HS hexasaccharide showed a shift towards the more compact conformation. These results led the authors to conclude that HS may cause a conformation change in hRobo1-Ig1-2. Such a change could easily facilitate either Slit2 binding or the propagation of a signal to the cell interior. An important caveat of these results is that IM-MS is a gas phase technique, and the observed conformations may not accurately reflect the solution state of the protein.

To further investigate the possibility of an HS-induced conformation change of hRobo1-Ig1-2, we performed solution NMR spectroscopy. Our strategy involved an isotopic labeling approach, suitable for application to glycoproteins expressed in mammalian cells, that produced a sample with ^13^C in methyl groups of both alanine and valine^10^. Resonances from these labeled sites were assigned using a software tool designed to work with sparsely labeled systems^11^. Of special interest are pseudo-contact shifts (PCSs), which provide long-range angle and distance- dependent (r^−3^) changes in resonance positions (chemical shifts) that can report on interdomain geometry. To provide these shifts, a lanthanide-binding peptide sequence (LBP4) was engineered into the Ig2 domain of hRobo1. Similar sequences have been engineered into loop regions of several proteins, but our construct contained an additional disulfide bond to preserve both the ion-binding capability of the loop and the antiparallel beta strand geometry of the introduction site^12–14^. All NMR measurements were performed with and without Arixtra (fondaparinux), a highly sulfated, synthetic HS pentasaccharide that forms a 1:1 complex with hRobo1-Ig1-2. Comparison of the two sets of PCS measurements allowed us to determine that the protein experiences only a small conformation change, but possibly a restriction in interdomain motion upon binding Arixtra. We discuss these changes with respect to propagation of signals to the cell interior.

## Results

We set out to investigate the domain orientation of hRobo1-Ig1-2 using a construct containing our inserted lanthanide-binding amino acid sequence. A construct incorporating a similar lanthanide-binding peptide (LBP) into a loop in the Ig1 domain was previously used to study ligand binding of a model HS tetrasaccharide^8^. This construct proved unsuitable for interrogating the domain orientation as few PCSs were observed from Ig2-domain residues. This was likely due to a poor choice of position, but we also suspected a high degree of loop motion and relatively low lanthanide binding affinity. Hence, we designed a new construct better suited to our purpose.

**Figure 1:**
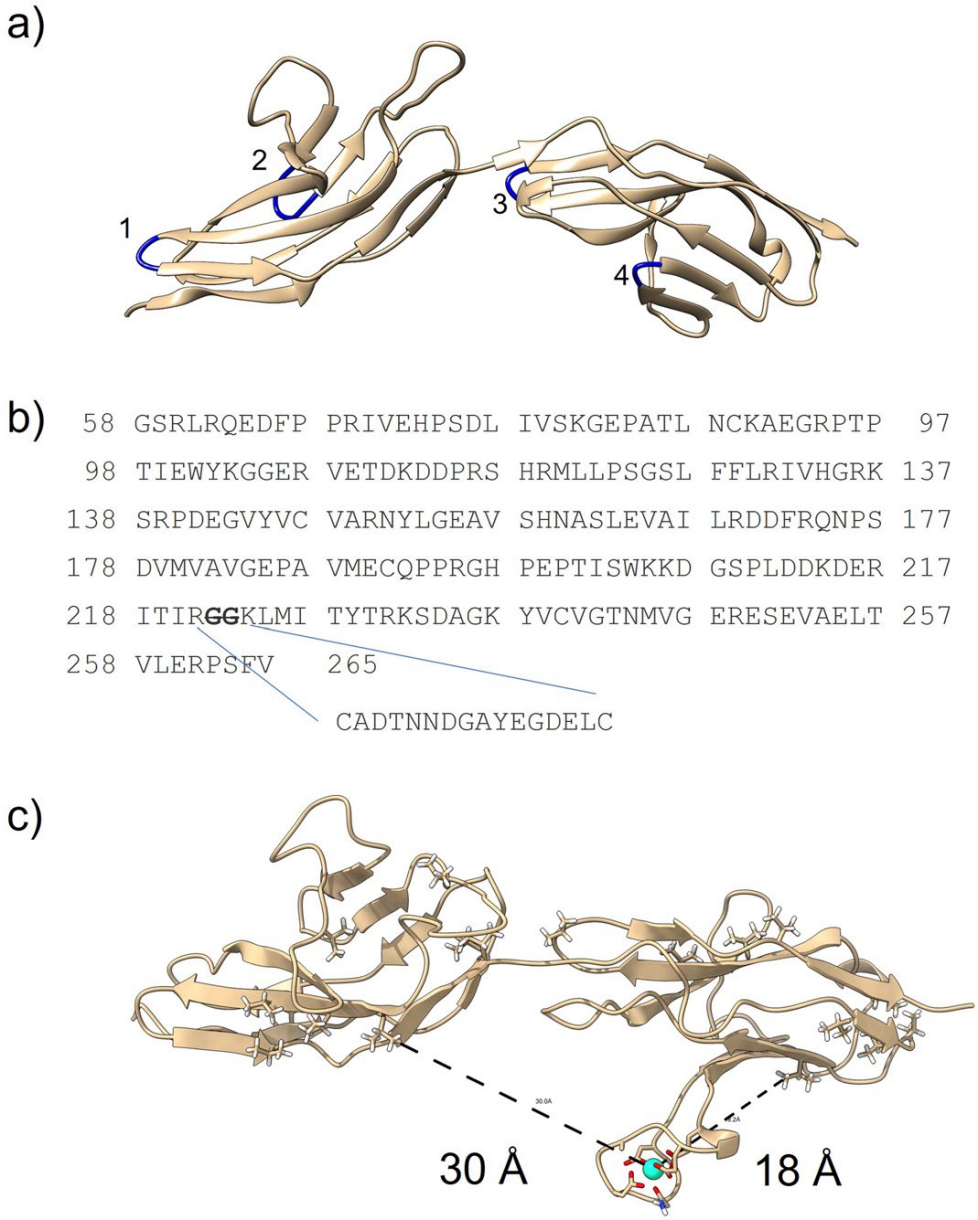
Design of hRobo1-Ig1-2-LBP4. (**a**) Structure of native hRobo1-Ig1-2 (PDB 2V9R). The four sites considered for LBP insertion are colored blue and labeled 1 through 4. (**b**) The modelled sequence of hRobo1-Ig1-2 is shown with the inserted lanthanide-binding sequence. Amino acid numbers from Uniprot entry Q9YN67 are used. (**c**) Modelled structure of hRobo1-Ig1-2-LBP4 taken from a cMD simulation. The dashed lines show distances between the modelled Dy^3+^ ion and two valine methyl groups, one in each domain, which suggests the loop is well positioned to inform on the domain orientation.

An ideal construct would contain the LBP in a position that places ^13^C-labeled sites in both domains within the ~ 30 Å sphere of PCS influence. A previous study showed that lanthanide sequences could replace β-turn motifs in interleukin-1β with minimal perturbation of the native structure^15^. Inspection of a hRobo1-Ig1-2 X-ray crystal structure found 4 candidate locations for inserting the LBP (Fig. 1). Both turns in the Ig1 domain face away from Ig2, which would place the lanthanide too far to produce significant PCSs in the Ig2 domain. The turn between Ig2 sheets F and G faces towards the Ig1 domain but is very close to the domain-domain interface. Insertion at this position could perturb the domain orientation and was not considered further. Lastly, the short turn between Ig2 sheets D and E also faces the Ig1 domain but is far enough away to accommodate insertion of the LBP without impacting domain orientation and was selected for these studies.

To stabilize the binding loop and minimize possible loop motion, we introduced cysteines at both ends of the inserted sequence, which we now refer to as LBP4. Formation of a disulfide bond between ends would stabilize the structure. Stabilization of the LBP in this way had been suggested previously and even a version omitting the original isoleucine, as in our LB4 sequence, had been tested and found to retain lanthanide binding affinity^16^. However, it was not clear that insertion at the ends of antiparallel β-strands had been tested. Fortunately, there is data suggesting that β_A,NHB_ sites (alternate positions in the β-strands) were compatible with disulfide bond formation^17^. Hence, we selected the pair of sites (222, 223 in the Uniprot sequence, Q9Y6N7) for insertion. Addition of cysteine residues to a protein construct can complicate protein expression and purification, often requiring refolding procedures for proper disulfide bond formation when expressing products in *E. coli* culture. However, in our case, mammalian cell cultures properly form disulfide bonds without further processing.

As an initial test of this proposed hRobo1-Ig1-2-LBP4 construct, an atomic model was built, and a long (1 µs) molecular dynamics (MD) simulation was performed. A bound dysprosium (Dy^3+^) ion was included in the model and remained in the binding site for the entire trajectory. The simulation also showed minimal disruption of the Ig2 fold, as the beta sheet between strands D and E remained intact. Comparison of 100 ns segments of trajectories on Robo1 constructs with and without an LBP also confirmed that interdomain motions were not significantly impacted by the presence of the LBP.

**Figure 2:**
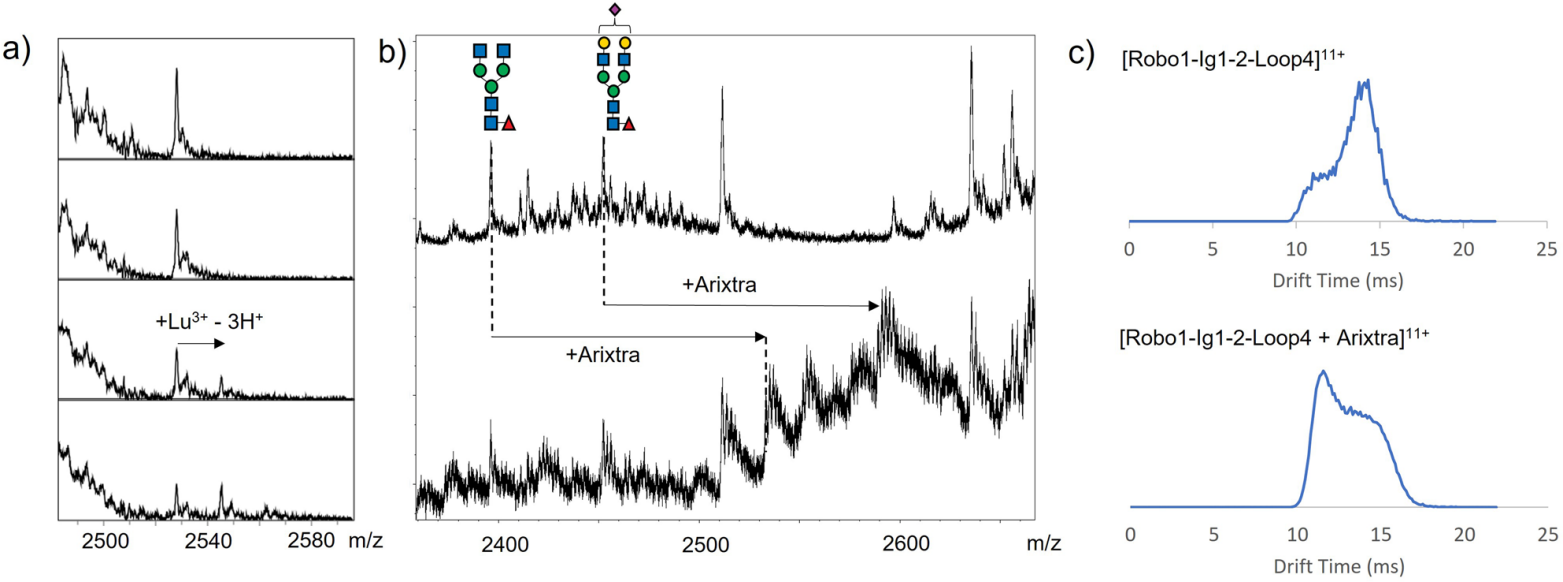
Native MS of hRobo1-Ig1-2-LBP4. (**a**) Titration with LuCl_3_. Panels show the same region of the native mass spectrum with increasing levels of lanthanide (0, 10, 20, and 30 µM from top to bottom). A peak consistent with the lanthanide-bound complex is observed at 20 µM LuCl_3_ and higher. (**b**) Comparison of native mass spectra without addition of Arixtra (top) and with an equimolar addition (bottom). Several new peaks appear in the presence of Arixtra. Protein-Arixtra complexes are indicated for two glycoforms. A plausible glycan consistent with the molecular weight is shown for each species. (**c**) Comparison of representative ion mobility arrival time distributions of a single glycoform alone and in complex with Arixtra. In both cases two peaks are observed corresponding to compact and extended conformations of the molecule. The distribution shifts toward the compact form in the protein-Arixtra complex.

A test batch of hRobo1-Ig1-2-LBP4 was initially expressed in HEK293F cells, and native mass spectrometry (MS) on a 10 µM sample was used to verify lanthanide binding activity. The spectra showed a heterogeneous mixture of complex-type glycoforms due to the single N-glycosylation site present in the Ig1 domain. Addition of lanthanide further increased the complexity of the spectra, but several glycoforms were sufficiently well-resolved to observe lanthanide binding. Figure 2a shows the results for a single glycoform on titrating our hRobo1-Ig1-2-LBP4 construct with lutetium chloride. After addition of 2 molar equivalents of lanthanide, the protein + Lu^3+^ complex is observed; with 3 molar equivalents roughly equal proportions of bound and unbound species are observed. Based on the results at 10 µM protein concentration, we hypothesized that at the elevated concentrations used for NMR (~ 300 µM), the lanthanide affinity would be sufficient for full complexation near 1:1 lanthanide to protein ratios. Furthermore, the MS data likely underestimate the affinity due to competition with formation of lutetium-acetate complexes in the ammonium acetate native MS solution.

Native IM-MS was also used to verify that our lanthanide-binding construct maintains heparan sulfate binding ability. To this end, we tested the construct’s ability to bind Arixtra (fondaparinux), a pentasaccharide drug reproducing a sulfation pattern found in heparin. In Fig. 2b a comparison of native mass spectra with (bottom) and without (top) an equimolar addition of Arixtra is shown. Several new species are observed upon addition of Arixtra, which are consistent with a 1:1 hRobo1-Ig1-2-LBP4/Arixtra complex for the various glycoforms. Considerable Na^+^/H^+^ heterogeneity is observed after the addition of Arixtra, which is typical of mass spectra of samples mixed with a highly sulfated oligosaccharide, for example as observed previously for heparan sulfate complexes with Robo1^9^. Nevertheless, these species are resolved sufficiently to allow the definitive assignment of a 1:1 complex of Arixtra with the hRobo1-loop construct. In the top spectrum, the two most abundant species have masses of 26,345 ± 2 and 26,963 ± 1 Da which agrees with hRobo1-Ig1-2-LBP4 GlcNAc_4_Hex_3_Fuc_1_ and GlcNAc_4_Hex_5_SiaFuc glycoforms (glycan composition only). In the bottom spectrum, new peaks appear with a mass increase of 1509 ± 2 Da, which agrees with that of Arixtra (C_31_H_53_N_3_O_49_S_8_). These results confirm that the lanthanide-binding construct retains HS binding activity.

Additionally, ion mobility data were collected. Travel of ions through a drift tube field with low pressure N_2_ gas is impeded in a manner dependent on cross-sectional area, and hence the conformation of ions^18^. Example arrival time distributions are shown in Fig. 2c. For both bound and unbound species, two features were observed which can be interpreted as a compact and extended conformation of the ion. The collision cross-sections (CCSs) derived from these measurements are 2110 Å^2^ for the feature with the early arrival time and 2383 Å^2^ for the feature with the longer arrival time. For the unbound state, the extended conformation was the more abundant feature, while for the bound form the distribution shifted towards faster drift times and a more compact conformation. The CCS values and the shift in arrival time are consistent with prior observations on an unmodified hRobo1-Ig1-2 construct and further validates use of this lanthanide-binding construct going forward^9^.

Having confirmed both lanthanide and HS-binding activity of our hRobo1-Ig1-2 construct, we expressed an isotope-labeled sample for NMR spectroscopy. The expression medium was enriched with ^13^C1-glucose and ^13^C-dimethyl-valine, which leads to ^13^C incorporation in all alanine and valine methyl groups^10^. NMR spectra were recorded on samples prepared with and without paramagnetic Dy^3+^ and with and without Arixtra (4 samples in total) allowing measurement of PCS data in bound and unbound states. Resonance assignments were determined using a custom MATLAB script based upon the Assign_SLP approach^11^. Briefly, resonances were assigned by comparison of measured and predicted data, including chemical shifts for H_γ_, C_γ_, H_β_, and H_α_ nuclei obtained from 3D-HSQC-TOCSYspectra and PCSs for Hγ, and Cγ methyl nuclei obtained from ^13^C-detected 2D-HSQC spectra. Using a genetic algorithm, the optimal assignment was found. Additional information is provided in the “Materials and methods” section.

**Figure 3:**
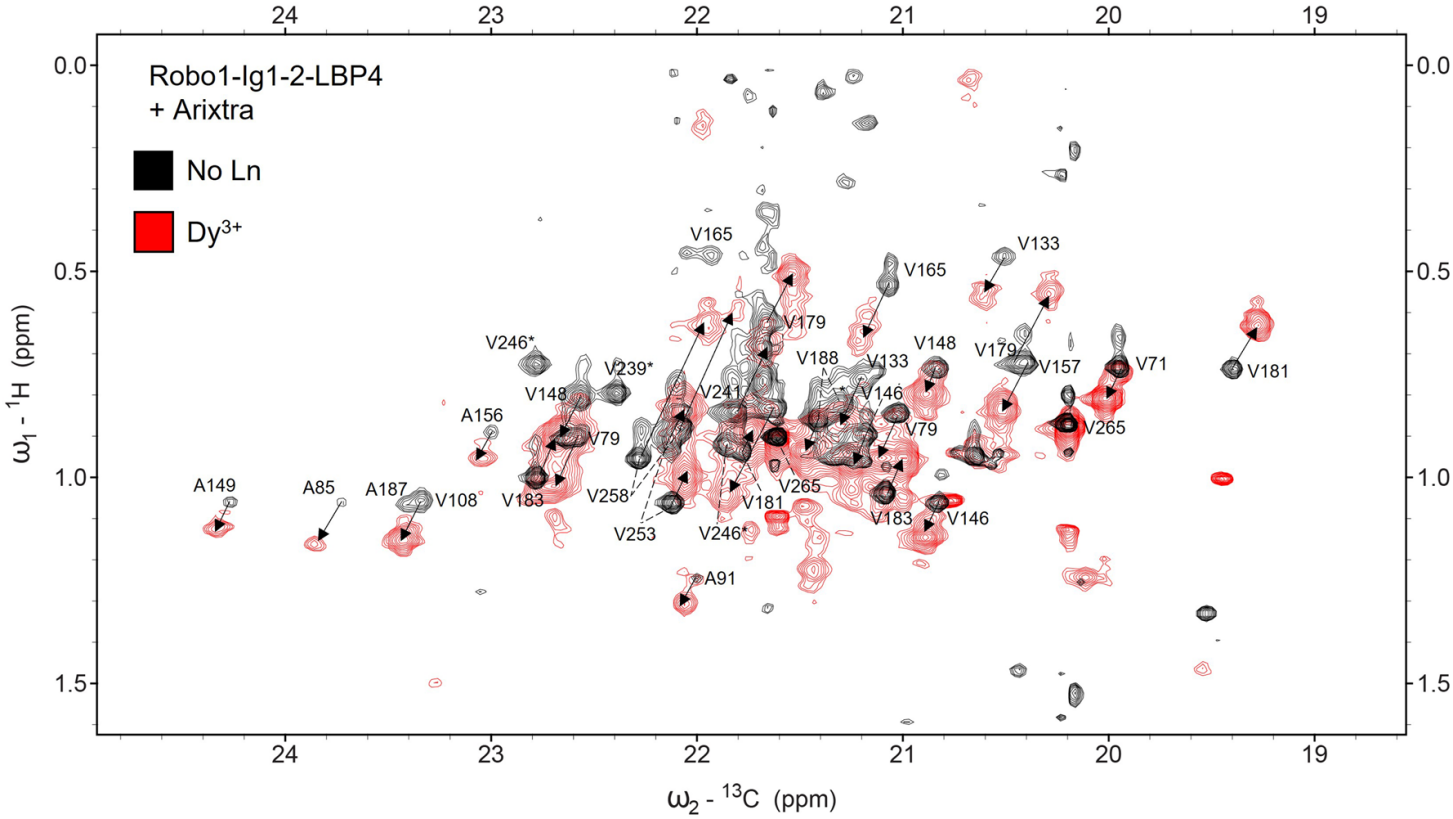
Overlay of HETCOR spectra of hRobo1-Ig1-2-LBP4 + Arixtra with (red) and without (black) Dy^3+^. The black arrows connect diamagnetic and paramagnetic peaks for the same resonance. Peaks are labeled next to their diamagnetic positions and in crowded regions are indicated by dotted lines. Several signals disappear in the Dy^3+^ spectrum and are indicated by asterisks. An overlay in the absence of Arixtra is included in Supplementary Fig. S1 and extracted data are summarized in Supplementary Tables S1 through S7.

**Figure 4:**
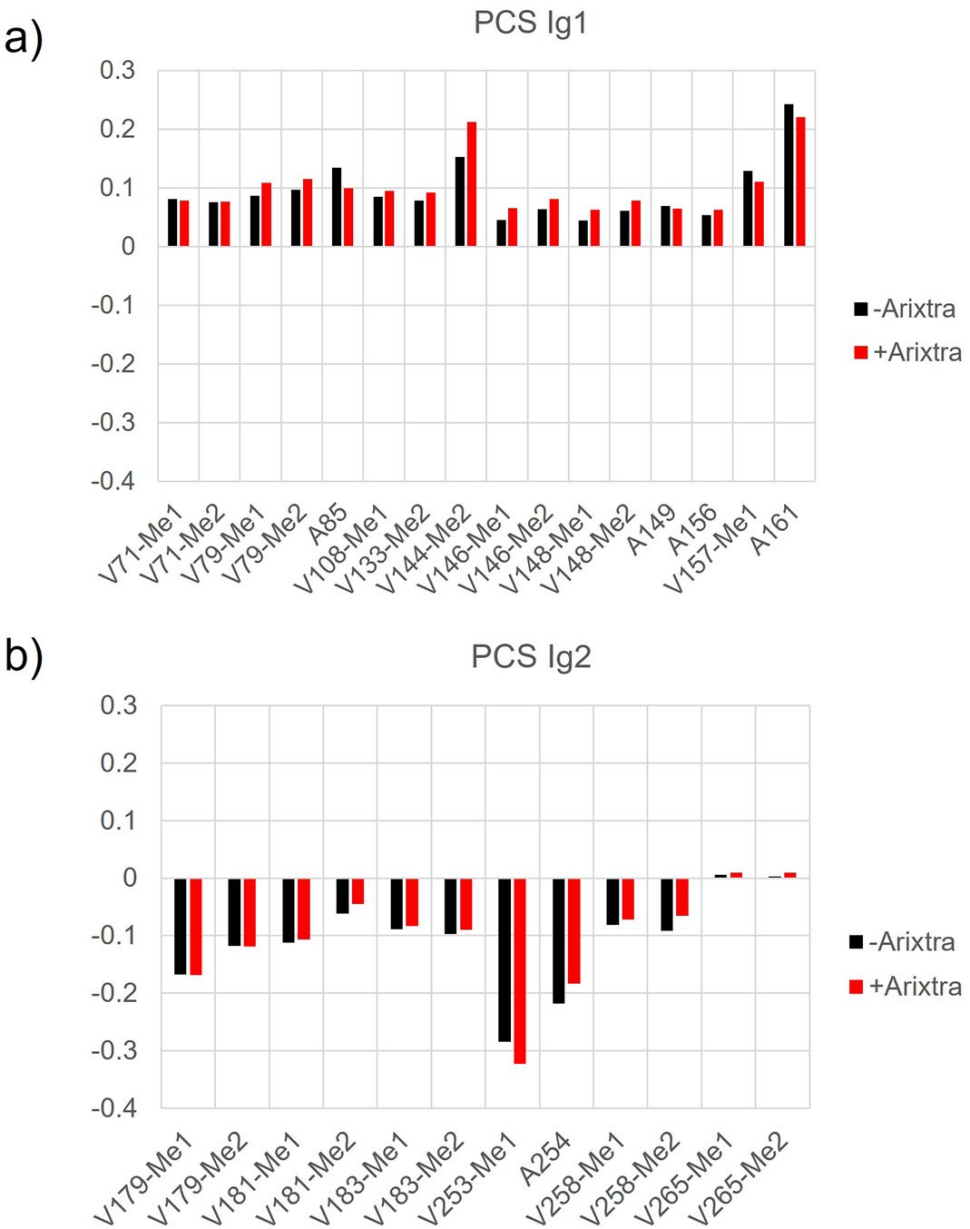
Comparison of Ig1-2 PCS measurements with (red) and without (black) Arixtra. Only those residues observed in both sets of data are shown. Signals from the Ig1 domain are shown in panel (**a**), while those from domain Ig2 are shown in panel (**b**).

PCS values were measured from ^13^C,^1^H-HETCOR spectra (Fig. 3). Direct observation of carbon nuclei proved useful for hRobo1-Ig1-2 by maximizing the resolution in the ^13^C dimension which accounted for most of the dispersion in the observed methyl signals. While peak overlap increases in the paramagnetic spectra, the majority of PCSs could be measured unambiguously by exploiting the 1:1 relation among shifts in each dimension. Several paramagnetic peaks were observed with no obvious diamagnetic counterpart and were not included. Comparison of PCS values measured with and without Arixtra show several changes (Fig. 4). Valine and alanine methyl groups in the Ig2 domain, where the LBP is located, showed an average change in PCS of 0.013 ppm (Fig. 4b). Large changes were not expected for this domain, but some of the largest changes are for V253, A254 and V258, all of which are on the exposed C-terminal strand, where they may be structurally perturbed by Arixtra binding near the strands’ N-terminal end.

Ig1 signals showed a larger change in their PCS values with an average of 0.018 ppm (Fig. 4a). V144-Me2 showed the largest change in PCS from 0.148 to 0.218 ppm. While some of these changes might be due to local effects due to Arixtra binding, the large change for V144-Me2 and the general pattern of larger changes in the domain removed from the lanthanide site suggests a change in the domain orientation.

The measured PCS data was next used to find the model of the domain orientation that best fits the data. Models were generated using Gaussian accelerated Molecular Dynamics (GaMD)^19^. Frames from the trajectory were clustered based on structural similarity, and the representative frame from the most probable cluster was chosen as an initial model. This model was similar to the X-ray structure 2V9R, having an RMSD over alpha carbons of just 2.1 Å. A grid search of possible conformers was then conducted by rotating the Ig1 domain, while leaving the Ig2 domain static. The pivot point for the rotations was chosen as the alpha carbon of isoleucine 165 which is centered in the linker between domains. Euler angles relative to a reference frame taken from the PDB 2V9R X-ray structure were then searched from − 90°to 90° for α and γ, while β was searched from 0° to 180°. Each conformer was scored against the PCS data by solving for the magnetic susceptibility tensor using the combined Ig2-domain measurements from both Arixtra-bound and unbound samples. Since the Arixtra-binding site is located primarily on the Ig1 domain, the presence of bound ligand is not expected to change the geometry of the lanthanide-binding tag and most other labeled residues on domain 2. Ig1-domain PCS values were then back-calculated using this tensor. The agreement between observed and back-calculated Ig1 PCS was assessed using a Q factor (Eq. (3), “Materials and methods”). Lower values of Q indicate better agreement between a structure and the PCS data.

**Figure 5:**
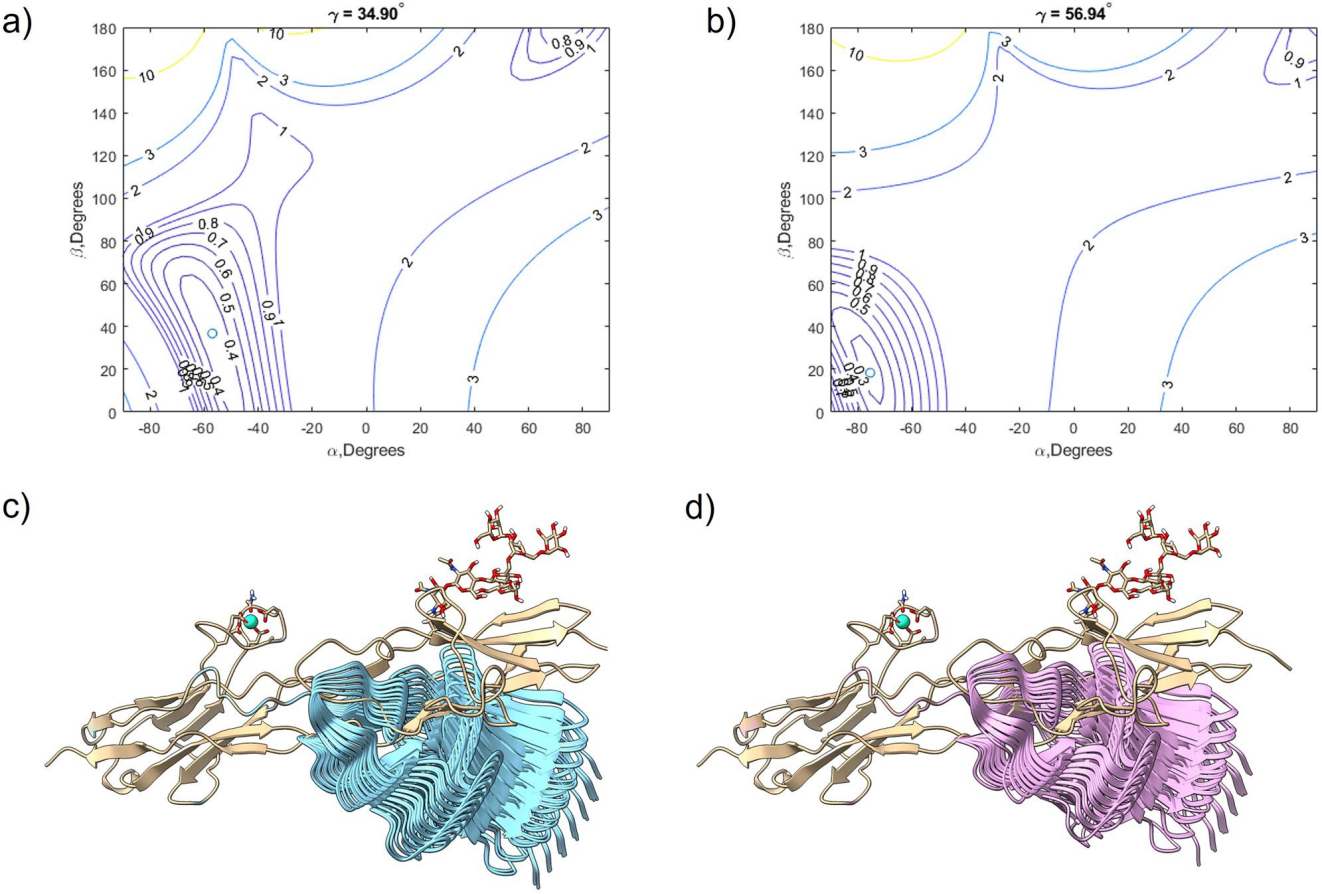
Results of Ig1 orientation grid search. (**a**) A contour plot showing the value of Q calculated using PCS data for unbound hRobo1-Ig1-2-LBP4 as a function of Euler angles α and β while γ is held constant at its optimum value. The best model is indicated as an open circle. (**b**) A second contour plot showing the value of Q calculated using PCS data for Arixtra-bound Robo1-Ig1-2-LBP4. (**c**) Overlay of the initial Robo1-Ig1-2-LBP4 conformation (tan) with the 20 lowest scoring models (cyan) found with unbound PCS data. (**d**) Overlay of the initial Robo1-Ig1-2-LBP4 conformation (tan) with the 20 lowest scoring models (pink) found with Arixtra-bound PCS data.

The results of the orientational grid search using the PCS data measured on the unbound form of hRobo1-Ig1-2-LBP4 are shown in Fig. 5a. The initial structure had a Q factor of 0.63 for Ig1 data, indicating poor agreement with the PCS data. After optimization this improved to a Q factor of 0.32 (Supplementary Fig. S3). The best orientation was obtained with Euler angles of − 56.9°, 36.7°, and 34.9° for α, β, and γ, respectively. Comparison of the top 20 best fitting Ig1 orientations with the starting structure shows an Ig1 orientation that is significantly different from the starting structure (Fig. 5c). These 20 models all fit the data quite well with small variations in Q and fall along a plane of orientations. While it is tempting to conclude that the spread of structures reflects underlying molecular motion, this is not necessarily true. More likely, the PCS data and the arrangement of valine methyls groups does not create a strong constraint on the orientation along the plane of orientations shown in Fig. 5c.

This process was repeated using the PCS data measured on the Arixtra bound state (Fig. 5b). The starting structure also showed poor agreement to the Ig1 PCS data, with a Q value of 0.72. The grid search found a slightly different Ig1 orientation to be most consistent with the PCS data, and a lower Q score of 0.25 (Supplementary Fig. S4). In this case the optimal Euler angles were − 75.3°, 18.4°, and 56.9 for α, β, and γ. The 20 lowest scoring conformers are shown in Fig. 5d, which adopt a similar orientation as that observed with the unbound data; however, in this case the bundle of Ig1 orientations is more tightly clustered. In this case, the lower Q factor indicates that the PCS data better fits a single rigid structure. The reduced spread of the 20 best models is a reflection of the deeper “well” of Q values observed in the contour plot (5B).

## Discussion

Interestingly, the structures which best fit our PCS data show a significant deviation from previously determined X-ray crystal structures. Crystal packing forces may be responsible for the bulk of differences in domain orientation observed in solution. The relatively small changes in PCS values upon binding Arixtra translate to a small, but significant, change in the predicted orientation of the Ig1 domain relative to the Ig2 domain. This highlights the sensitivity of PCS data to small structural changes. However, it is important to note that this analysis was performed using a single structure and does not include the effects of averaging over multiple conformations. Our MD simulations indicate that hRobo1-Ig2 exhibits considerable flexibility and likely exists in multiple states in solution. As such, our PCS results reflect changes in the domain orientation averaged over an ensemble of conformers. Nonetheless, the improvement in Q score for the Arixtra-bound PCS dataset suggests that the flexibility has decreased. HS is known to bind near the hinge region connecting the two domains, and thus it would not be surprising that the presence of a ligand would limit the extent of interdomain motion.

While the NMR data suggests a slightly more bent hinge region between the two domains of the protein than was observed by X-ray crystallography, the hinge region is considerably less bent than was inferred from the ion mobility measurements that motivated this work^9^. This discrepancy is likely the result of gas-phase compaction of hRobo1-Ig1-2. Experimental collision-cross sections (CCS) of globular proteins are often found to be smaller (~ 10%) than predictions from X-ray or NMR structures^20^. This small degree of compaction is often attributed to “self-solvation” and rearrangement of surface side-chains. More extreme deviations in CCS values have been observed for non-globular proteins and proteins containing flexible linkers (e.g. IgG antibodies)^21^. While hRobo1-Ig1-2 does not have a long disordered linker between domains, there may be sufficient flexibility to collapse into a less extended structure upon transfer to the gas phase. While the gas-phase conformation of hRobo1-Ig1-2 may exaggerate the bend of the hinge region, it does point in the direction of a more bent conformation than the crystal structure.

The finding that Robo1-1g1-2 is slightly more bent than initial crystal structures and that HS decreases the flexibility about the domain junction, may have some implications for the mechanism of Robo-Slit signaling. Recent studies on larger Robo1 constructs have focused more on homotypic Robo1 interactions than Slit2 and HS binding. They show that Robo1 forms dimers and tetramers that exist in active and inactive conformations^6,22^. Dimerization appears to be driven primarily by interactions between Ig4 domains and the role of Slit2 is suggested to be disruption of oligomeric states. However, the relative orientations of Robo1 Ig1 and Ig2 domains in new crystal structures^6^ is very similar to that of the 2V9R crystal structure^5^. HS is also not included in the structures and the only high-resolution data on the Slit2-Robo1 interaction remains that from a crystal structure that includes only the first Robo1 domain, 2V9T^5^. Our results rule out any large HS induced change in Ig1-Ig2 interdomain orientation. However, it is possible that the small changes we do observe could directly enhance Slit-Robo1 binding. It is also possible that the restriction in interdomain motion could enhance binding through entropic effects. HS-binding may also have more long-range effects. It could simply facilitate diffusion of Robo1 and Slit2 within the extracellular matrix. Diffusion along a one-dimensional HS “rail” would be much more efficient than in three dimensions and aid the two proteins finding one another. HS may also strengthen the interaction between the two proteins by bridging both proteins. Further structural studies incorporating HS, extended Robo1 constructs, and Slit2 would be necessary to address these hypotheses.

The methods we have described in this report can play important roles in future structural studies. Sparse isotopic labeling of methyl groups is possible in a wide range of cell cultures, including those that provide native types of glycosylation^10^. NMR of methyl groups also provides the relatively high sensitivity and resolution needed for the study of larger structures^23^. When combined with paramagnetic lanthanide tags, numerous long-range structural restraints can be measured. These can easily span the range necessary for the study of protein–protein and protein-glycosaminoglycan complexes. The present application depended on previously determined X-ray crystal structures for NMR resonance assignment and initiation of accelerated MD studies. However, recent advances in the accuracy of structure prediction algorithms^24^, suggest that computational models may suffice. This would greatly increase opportunities for the application of methods described.

## Materials and methods

### Protein expression and purification

The hRobo1-Ig1, hRobo1-Ig1-2, and hRobo1-Ig1-2-LBP4 constructs were synthesized using codons optimized for mammalian cell expression by GeneArt (Regensburg, Germany). The one-domain construct comprised residues 61 to 169 (Uniprot Q9Y6N7), while both two-domain constructs comprised residues 61 to 266. The lanthanide-binding loop was derived from the original Imperiali construct by replacement of amino acids at both ends with cysteines, deletion of an isoleucine group and replacement of a tryptophan with an alanine^15^. This resulted in the amino acid sequence CADTNNDGAYEGDELC, which was inserted between residues R221 and K224 while residues G222 and G223 were deleted (numbering from Uniprot entry Q9Y6N7). The constructs were inserted into pGEn2 expression vectors^25^. The vectors contained codons for an N-terminal hexahistidine-tag and GFP protein separated by a short linker and TEV cleavage site.

Both protein expression and purification proceeded as previously described^13,26^. Cleavage with TEV left a short scar on the N-terminus of the product (GSGG). A preliminary expression was performed in HEK293F cells, while a second isotope-labeled batch was expressed in HEK293S (GnT1^−^, MGAT1 knockout) cells (ATCC), which add primarily Man_5_GlcNAc_2_ glycans to N-glycosylation sites. Growth and expression of this batch proceeded in 500 mL of a custom version of FreeStyle 293 expression media (Gibco, ThermoFisher Scientific) which lacked both glucose and a select set of amino acids, namely lysine, phenylalanine, tyrosine, and valine. To this medium we added 2.5 g of ^13^C1-glucose and 75 mg of ^13^C-methyl-valine (Cambridge Isotope Labs, Tewksbury, MA), along with supplied supplements of lysine, phenylalanine and tyrosine. After two rounds of metal affinity chromatography and a round of size exclusion chromatography, the final yield was ~ 10 mg. Protein concentration was determined by UV/Vis absorbance measurement at 280 nm. An extinction coefficient of 20,315 M^−1^ cm^−1^ was predicted using the ProtParam tool on the Expasy webserver and used in the calculations^27^.

### Native mass spectrometry

Purified hRobo1-Ig1-2-LBP4 from HEK293F cells was buffer exchanged into 10 mM ammonium acetate, pH 6.8 using Amicon microconcentrators (10 kDa molecular weight cutoff) for native MS and IM-MS measurements. Samples were immediately frozen and stored at − 20 °C prior to analysis.

Assessment of lanthanide binding was performed using a 12 T Bruker Solarix FT-ICR-MS instrument. The instrument was calibrated for high-m/z using 1 mg/mL sodium perfluoroheptanoate (Sigma-Aldrich) in 50:50 (v/v) % acetonitrile/water. Samples were prepared with 10 µM protein and 0, 10, 20, and 30 µM of LuCl_3_. Samples were infused by syringe pump at a rate of 2.0 µL/min and ionized via electrospray (ESI) at a voltage drop of 4500 V. Ions were desolvated with a skimmer 1 voltage of 100 V and accumulated in the collision cell for 1.0 s prior to injection into the ParaCell, where ions were trapped after a 1.4 ms time of flight delay. Ions were excited for broadband detection by a chirp waveform at 40% excitation power. Spectra were collected from m/z 500 to 5000 with 1 M points and a transient duration of 1.4 s. Each spectrum was the sum of 100 scans. Spectra were analyzed using Bruker DataAnalysis software.

Ion mobility mass spectrometry data were collected using a Waters Synapt G2-S instrument. The protein concentration was 10 µM. hRobo1-2-LBP4 + Arixtra spectra were collected on a solution prepared with an additional 10 µM Arixtra (fondaparinux sodium salt, Sigma-Aldrich). Solutions were directly infused at flow rates of 0.5–1.0 µL/min. ESI was achieved using fused silica emitter tips (I.D. 15 um, new objective) and a Waters Zspray source operating at capillary voltages of 1.8–2.5 kV. To ensure gentle ionization conditions the source block temperature was set to 30 °C, the sampling cone was kept at 30 V, and the extraction cone was set to 1.0 V. The traveling wave in the IMS cell had a velocity of 300 m/s and an amplitude of 21 V. IM-MS data was exported to a csv file using TWIMExtract^28^. Collision cross-sections were derived from arrival times, based on calibration with protein standards as described previously^9^.

### NMR spectroscopy

NMR spectroscopy was performed on ^13^C1-Glucose, ^13^C-dimethyl-valine, labeled hRobo1-Ig1-2-LBP4. The protein was initially exchanged into a buffer composed of 25 mM Tris, 100 mM NaCl, pH 7.4, 0.02% NaN_3,_ 10 µM DSS, 90/10% H_2_O/D_2_O at a final protein concentration of 300 µM. For PCS data a near molar equivalent (~ 0.9) of DyCl_3_ was added. Addition of diamagnetic LuCl_3_ lead to prohibitive levels of protein precipitation and a sample containing no lanthanide was used as a reference instead. For Arixtra binding experiments, Arixtra was titrated to a final concentration of 600 µM.

NMR spectra were acquired on a Bruker AVANCE NEO 900 MHz spectrometer using a triple resonance TXO cryo probe that is optimized for ^13^C and ^15^N observation. ^13^C, ^1^H-HETCOR spectra were recorded with 2048 × 192 points using the standard Bruker hxinepph sequence. Sweep widths of 61 ppm and 3.0 ppm were used for the ^13^C and ^1^H dimensions, respectively. INEPT delays were set to 1/(4 J) (2.0 ms), while the refocusing delays were set to 1/(12 J) (0.667 ms), where J was assumed to be 125 Hz. 96 scans were averaged. All spectra were processed using nmrPipe^29^. Peak picking was performed with NMRFAM-Sparky^30^.

3D-^13^C-edited-HSQC-TOCSY data were collected identically on 300 µM samples of both hRobo1-Ig1 and hRobo1-Ig1-2 constructs using the mlevhsqcetgp3d pulse sequence and recorded with 2048, 64, and 128 points in t_3_, t_2_, and t_1_ dimensions, respectively. The indirect dimensions were sampled with a 5% NUS scheme generated within TopSpin. A TOCSY mixing time of 60 ms was employed. The resulting spectra were processed in nmrPipe and reconstructed using the SMILE algorithm^29,31^.

### Resonance assignment

Resonance assignments for valine methyl groups were made by initially focusing on a truncated hRobo1-Ig1 construct. The remaining valine methyl groups were then assigned to the Ig2 domain using a hRobo1-Ig1-2 construct without an inserted lanthanide binding loop. Assignments were further constrained by mutagenesis of six of the valines (V71, V133, V165, V188, V241 and V246) isoleucine. In both cases remaining assignments were determined using a custom script based on the Asssign_SLP program^11^. Experimental C_γ_, H_γ_, H_β_, and H_α_ chemical shifts measured from 3D-HSQC-TOCSY spectra were compared with predicted values determined using ShiftX2 (Table S1)^32^, while mutagenesis information was included as constraints on the scoring function. Crosspeak assignments were readily transferred to the hRobo1-Ig1-2-LBP4 construct. The assigned valine methyl resonances were used to assist assigning alanine methyl signals by comparing their PCS values.

### Model building and molecular dynamics simulations

A model of hRobo1-Ig1-2-LBP4 was built starting from an available X-ray crystal structure (PDB 2V9R) using UCSF chimera^5,33^. Initial coordinates for the LBP were taken from the X-ray crystal structure of interleukin-1β with a lanthanide-binding insertion sequence (PDB 3LTQ, residues 53A through 53P) and modified to match our modified construct^12^. Phi and psi angles near the fusion were adjusted to smoothly extend the beta sheet. Hydrogen atoms were added with the reduce tool^34^. Aspartic acid residue 97 was changed to the native asparagine and a Man_5_GlcNAc_2_ glycan was added using tleap^35^.

The system for a conventional molecular dynamics (cMD) simulation was prepared and run using Amber2018 with the ff99SB forcefield for amino acids and Glycam-06j forcefield for carbohydrates. The hRobo1-Ig1-2-LBP4 model was solvated with a truncated octahedron of TIP5P water and neutralized by the addition of sodium cations^36^. The resulting system was energy minimized with the Sander module using 25,000 steps of steepest descent minimization followed by 46,996 steps of conjugate gradient minimization. The system was slowly heated to 300 K over 1 ns and then the density was equilibrated for 1 ns in the NPT ensemble. The system was further equilibrated by 50 ns of NVT simulation. The production cMD run consisted of 1 µs of NVT simulation.

A gaussian accelerated MD (GaMD) simulation was performed starting from the same equilibrated system with randomized initial velocities^19^. Acceleration was performed with a dual-boost scheme applied to both the total potential and the dihedral potential. Energy statistics were calculated from an initial 14 ns NVT simulation and both ${\sigma }_{0D}$ and ${\sigma }_{0P}$ were set to 6.0. Likely conformers were selected by clustering the frames via their C_α_ RMSD in MATLAB using functions from MDToolBox^37^. Clusters containing more than 300 frames were chosen for energy reweighting, which was also performed in MATLAB using the approach described by Miao et al.^19^.

### PCS analysis

PCS data were fit to PDB structures using custom MATLAB code. The magnetic susceptibility tensor was determined from the following linear system of equations:

1 $$M\Delta \chi =P$$

where M is a n × 5 matrix with row corresponding to a single atomic nucleus, Δχ is a 5 × 1 matrix containing five independent elements of the anisotropic part of the magnetic susceptibility tensor, and P is a n × 1 matrix containing the PCS values measured in ppms. The elements are constructed from the cartesian coordinates of the ion-nucleus vector as follows, where *r* is the vector length in meters:

2 $$M=\frac{1}{{12\pi r}^{5}}\left[\begin{array}{ccccc}\frac{1}{2}\left({2z}^{2}-{x}^{2}-{y}^{2}\right)& \frac{1}{2}\left({x}^{2}-{y}^{2}\right)& 2xy& 2xz& 2yz\\ \vdots & \vdots & \vdots & \vdots & \vdots \end{array}\right]$$

With at least 5 PCS values, the above system can be solved for Δχ. This approach is quite similar to that used by REDCAT (albeit for RDC calculations) and Paramagpy^38,39^. Agreement between a given structure and the PCS measurements was assessed by calculating a Q-factor, which is given by Eq. (3).

3 $$Q=\frac{\sum {\left({PCS}_{obs}-{PCS}_{calc}\right)}^{2}}{\sum {\left({PCS}_{obs}\right)}^{2}}$$

### Ig1 orientational grid search

A grid search over possible Ig1 orientations was conducted using a MATLAB script. A starting structure was produced using the representative frame of the most probable cluster from the GaMD simulation. This frame was then aligned with an X-ray crystal structure (PDB 2V9R) via the Ig2 domain and saved relative to the X-ray coordinate system. Different orientations of the Ig1 domain were generated using Euler angle rotations (ZXZ convention) about the alpha carbon within residue Ile 104. For each Euler angle 50 values were used, creating a 3-dimensional grid of 125,000 possible orientations. Each orientation was scored by calculating a Q-factor (Eq. 3) for the Ig1 PCS data. The $\Delta \chi$ tensor used to back-calculate PCS values was determined from an Ig2 dataset made by combining data from both Arixtra-bound and unbound samples. The principal values of the tensor in m^3^ are: ${\Delta \chi }_{xx}=-4.3\times {10}^{-32}$, ${\Delta \chi }_{yy}=-34\times {10}^{-32}$, and ${\Delta \chi }_{zz}=38\times {10}^{-32}$.

## Supplementary Information

Supplementary Information.

## Data Availability

All data generated or analyzed during this study are included in this published article and its Supplementary Information files. Chemical shift assignments have been deposited in the Biological Magnetic Resonance Bank, accession number 51445.
