# Supplementary material for: NMR analysis suggests the terminal domains of Robo1 remain extended but are rigidified in the presence of heparan sulfate
Source: Sci Rep. 2022 Aug 30;12:14769. doi: 10.1038/s41598-022-18769-6 (PMC9427851; doi:10.1038/s41598-022-18769-6)
Supplement: Supplementary file 1 — Supplementary Information. [file 41598_2022_18769_MOESM1_ESM.pdf]

Supplemental Information for

NMR Analysis Suggests the Terminal Domains of Robo1 Remain Extended but are Rigidified in the Presence of Heparan Sulfate

Robert V. Williams, Chin Huang, Kelley W. Moremen, I. Jonathan Amster, James H. Prestegard*

MATLAB Code used for resonance assignment.

```
% Main script
% load data
expt = readtable('Robo1-Ig1_val_HB-CG-HA-HB_exp.xlsx');
pred = readtable('Robo1-Ig1_val_HB-CG-HA-HB_pred.xlsx');

nPeaks = size(expt,1);
nResidues = size(pred,1);

residues = pred{:,1};

% define weights for different chemical shifts
% order is CG, HG, HB, HA
w = [5,0.4,5,8];

% change 999 to nan
expt(7,5) = {NaN};


%% add constraints
constraints = ones(nPeaks);

% V71
constraints(:,[1,2]) = 0;
constraints([3,4],:) = 0;
constraints([3,4],[1,2]) = 1;

% V165
constraints(:,[17,18]) = 0;
constraints([1,2],:) = 0;
constraints([1,2],[17,18])=1;

% V144
constraints(:,[9 10]) = 0;
constraints([15,16],:) = 0;
constraints([15,16],[9 10])=1;
```

```
% add methyl pairing info
resPairExp = [1,1,2,2,3,3,4,4,5,5,6,6,7,7,8,8,9,9]';
resPairPred =
[71,71,79,79,108,108,133,133,144,144,146,146,148,148,157,157,165,165]'
;
%% set up GA search
populationSize = 1000;

FitnessFcn = @(x) myScore(x,expt,pred,w,constraints) +
residuePairPenalty(x,resPairExp,resPairPred);

options = optimoptions(@ga,'PopulationSize',populationSize, ...
    'PopulationType', 'custom',...

'CreationFcn',@(nPeaks,FitnessFcn,options)create_permutations(nPeaks,F
itnessFcn,options), ...
    'CrossoverFcn',@crossover_permutation,...
    'MutationFcn',@mutate_permutation, ...
    'Generations',200, ...
    'StallGenLimit',30,...
    'Vectorized','on', ...
    'EliteCount',.05*populationSize,...
    'CrossoverFraction',0.2, ...
    'PlotFcn',
@(options,state,flag)myPlot(options,state,flag,residues,nPeaks));

% run GA search
[x,fval,exitflag,output,finalPop,finalScores] = ...
    ga(FitnessFcn,nPeaks,[],[],[],[],[],[],[],options);

[score, subScore] = myScore(x,expt,pred,w,constraints);

%% Declare subroutines
function [totalScore,subScores] =
myScore(x,expt,pred,w,constraints)
%% scoring function for Chemical shifts
% Inputs
%   x       assignment vector, m x n cell
%           m assignments with n variables
%
%   expt    table of experimental data, n x 6 table
%
%   pred    table of predicted data, n x 6 table
%
%   w       weights for different shifts, 4 x 1 double array
%
% Outputs

penalty = 1;
```

```
m = size(x,1);
nPeaks = size(x{1},2);
totalScore = zeros(m,1);

subScores.CG = zeros(m,1);
subScores.HG = zeros(m,1);
subScores.HG = zeros(m,1);
subScores.HA = zeros(m,1);
subScores.Penalty = zeros(m,1);

for i = 1:m

    % rearrange experiment
    test = expt(x{i}',:);

    subScores.CG(i) = getRMSD(test.CGShift,pred.CGShift);
    subScores.HG(i) = getRMSD(test.HGShift,pred.HGShift);
    subScores.HB(i) = getRMSD(test.HBShift,pred.HBShift);
    subScores.HA(i) = getRMSD(test.HAShift,pred.HAShift);

    for j = 1:nPeaks
        if constraints(x{i}(j),j)==0
            subScores.Penalty(i) = subScores.Penalty(i) +
penalty;
        end
    end


    totalScore(i) = w(1)*subScores.CG(i) + w(2)*subScores.HG(i)
+ w(3)*subScores.HB(i) + w(4)*subScores.HA(i) +
subScores.Penalty(i);
end
end

function rmsd = getRMSD(x,y)

rmsd = sqrt(mean((x-y).^2,'omitnan'));

end

function penalty = residuePairPenalty(x,resPairExp,resPairPred)
% residuePairPenalty - check that intraresidue peaks are
assigned to the same residue

rows = size(x,1);
penalty = zeros(rows,1);

for i = 1:rows
```

```
    d = x{i};
    n = size(d,2);

    for j = 1:n
        % find all peaks constrained to be in same residue
        residueNum = resPairExp(d(j));
        idx = find(resPairExp == residueNum);

        % are predicted counterparts also intraresidue?
        idx2 = zeros(size(idx));
        for k = 1:size(idx,1)
            idx2(k) = find(d==idx(k));
        end
        test = resPairPred(idx2);
        if all(test == test(1))
            % yes - no penalty
        else
            % no - apply penalty
            penalty(i) = penalty(i) + 1;
        end
    end

end

end

function state =
myPlot(options,state,flag,residues,nMeasurements)
% ASSIGN_SLP_figure Plot heatmap of current GA population

if state.LastImprovement
    [~,I] = min(state.Score);
    bestAssign = state.Population{I};
    M = zeros(nMeasurements);

    for j = 1:size(state.Population,1)
        current = state.Population{j};

        for i=1:nMeasurements
            if bestAssign(i)==999
                bestAssign(i)=0;
            end

            M(current(i),i) = M(current(i),i) + 1;
        end
    end
```

```
    heatmap(residues,(1:nMeasurements),M);
    xlabel('residue from coordinate file');
    ylabel('peak');
    titleStr = sprintf('Generation %d, Best Score: 
%.3f',state.Generation,min(state.Score));
    title(titleStr);
end

end
```

MATLAB Code used for Ig1 orientation search. Identical scripts were used to analyze Arixtra-bound and unbound PCS data.

```
% load inputs
pcsTable = readtable('20220314_Robo1-Ig1-Ig2-
Loop4_PCS_AlaUpdate.xlsx');
pdb = readpdb('robo1-ig2man5-GaMD.fr167596.2v9rFrame.pdb');

% leave out some PCS that don't seem quite right
pcsTable.UnboundPCS_1(38) = NaN;
pcsTable.UnboundPCS_2(38) = NaN;
pcsTable.BoundPCS_1(41) = NaN;
pcsTable.BoundPCS_2(41) = NaN;
pcsTable.BoundPCS_1(38) = NaN;
pcsTable.BoundPCS_2(38) = NaN;
pcsTable.UnboundPCS_1(41) = NaN;
pcsTable.UnboundPCS_2(41) = NaN;

% V133 is an outlier, present in flexible loop may not be well 
represented
% in a single structure
pcsTable.UnboundPCS_1(10) = NaN;
pcsTable.UnboundPCS_2(10) = NaN;
pcsTable.BoundPCS_1(10) = NaN;
pcsTable.BoundPCS_2(10) = NaN;

idx_Ig1 = pdb.resseq < 104;
idx_Glycan = pdb.resseq > 212; % Glycan is in Ig1
idx_Ig2 = pdb.resseq >= 104 & pdb.resseq <= 212;

idx_Dy = selectname(pdb.name,'Dy');
xyz_Dy = pdb.xyz(idx_Dy,:);

idx_val_methyl = selectname(pdb.resname,'VAL') & 
selectname(pdb.name,'CG1','CG2');
```

```
idx_ala_methyl = selectname(pdb.resname,'ALA') &
selectname(pdb.name,'CB');
idx = idx_val_methyl | idx_ala_methyl;

Me_xyz = pdb.xyz(idx,:);
Me_xyz_pcs = Me_xyz - xyz_Dy;

ig2_rows = 24:48;
ig1_rows = 1:23;
[ig2_tensor,~,Q_ig2] =
PCScalc(Me_xyz_pcs(ig2_rows,:),pcsUnBound(ig2_rows),true);

%%
% set up rotation axis
% Ile 104 is in the hinge region between domains
% a vector between CA and CG1 would make a good rotation axis
ref = pdb.xyz(selectid(pdb.resseq,104) & selectname(pdb.name,'CA'),:);

npts = 50;
a = linspace(-pi/2,pi/2,npts);
b = linspace(0,pi,npts);
c = linspace(-pi/2,pi/2,npts);

%
Q_unbound = zeros(50,50,50);
Q_bound = zeros(50,50,50);
% crd = cell(50,50,50);
bc_unbound = cell(50,50,50);
bc_bound = cell(50,50,50);
for i = 1:50
    for j = 1:50
        for k = 1:50
        tmp = pdb.xyz(idx_Ig1,:);
        tmpGlycan = pdb.xyz(idx_Glycan,:);
        tmpGlycan = tmpGlycan - ref;
        tmp = tmp  - ref;

        tmp = eulerRotate(tmp,a(i),b(j),c(k));
        tmpGlycan = eulerRotate(tmpGlycan,a(i),b(j),c(k));

        tmp = tmp + ref; % translate back
        tmpGlycan = tmpGlycan + ref;

        xyz = [tmp;pdb.xyz(idx_Ig2,:);tmpGlycan];
        xyz_pcs = xyz - xyz_Dy;

        % use Ig2 tensor to back-calculate
        Me_xyz_pcs = xyz_pcs(idx,:);
        [~,bc_unbound{i,j,k},Q_unbound(i,j,k)] =
PCScalc(Me_xyz_pcs(ig1_rows,:),pcsUnBound(ig1_rows),false,ig2_tensor);
        [~,bc_bound{i,j,k},Q_bound(i,j,k)] =
PCScalc(Me_xyz_pcs(ig1_rows,:),pcsBound(ig1_rows),false,ig2_tensor);
```

```
        end
    end
end

%% plotting
[A,B] = meshgrid(a,b);
A = A*180/pi; % convert from rads to degrees
B = B*180/pi;
foldername = 'contour_figs_unbound_20220314';
for i = 1:50
     c_plane = i;
     contour(A,B,Q_unbound(:,:,c_plane)',[0.3, 0.4, 0.5, 0.6, 0.7,
     0.8, 0.9, 1, 2, 3,10],'ShowText','on')
     xlabel('\alpha,Degrees')
     ylabel('\beta,Degrees')
     title(['\gamma = ',num2str(c(i)*180/pi,'%.2f'),'^\circ'])
     % f = gcf;
     %filename = sprintf('contour_ab_g%d',i);
     %exportgraphics(f,[foldername,filesep,filename,'.png'],'Resolutio
     n',300)
     pause(0.1)
end

Q_unbound = permute(Q_unbound,[1,3,2]); % permuting again recovers
original

%% % find top 20 models
nModels = 20;
Q_best_unbound = min(Q_unbound(:));
[Q_sort,I] = sort(Q_unbound(:));
% get indices for Q
idx_1 = mod(mod(I,2500),50); % this is alpha
idx_2 = ceil(mod(I,2500)/50); % this is beta
idx_3 = ceil(I/2500); % this is gamma
idx_mat = [idx_1,idx_2,idx_3];

% do the rotation again
crd = zeros(nModels,3*3402);
for i = 1:nModels
tmp = pdb.xyz(idx_Ig1,:);
tmp = tmp  - ref;
tmpGlycan = pdb.xyz(idx_Glycan,:);
tmpGlycan = tmpGlycan - ref;

tmp = eulerRotate(tmp,a(idx_1(i)),b(idx_2(i)),c(idx_3(i)));
tmpGlycan =
eulerRotate(tmpGlycan,a(idx_1(i)),b(idx_2(i)),c(idx_3(i)));

tmp = tmp + ref; % translate back
tmpGlycan = tmpGlycan + ref;

xyz = [tmp;pdb.xyz(idx_Ig2,:);tmpGlycan];
```

```
xyz = xyz';
xyz = xyz(:);
crd(i,:) = xyz';
end

%% write as separate models in PDB file
outFolder = 'results';
outFile = 'bestModels_UnBoundData_top20_20220314.pdb';
% writepdb([outFolder,filesep,outFile],pdb,crd)

resultsTable = table((1:nModels)',...
    Q_sort(1:nModels),...
    a(idx_1(1:nModels))' * 180/pi,...
    b(idx_2(1:nModels))' * 180/pi,...
    c(idx_3(1:nModels))' * 180/pi,...
    'VariableNames',{'Rank','Q','Alpha','Beta','Gamma'});
writetable(resultsTable,'20220405_figures/20220406_top20models_results
.xlsx')
```

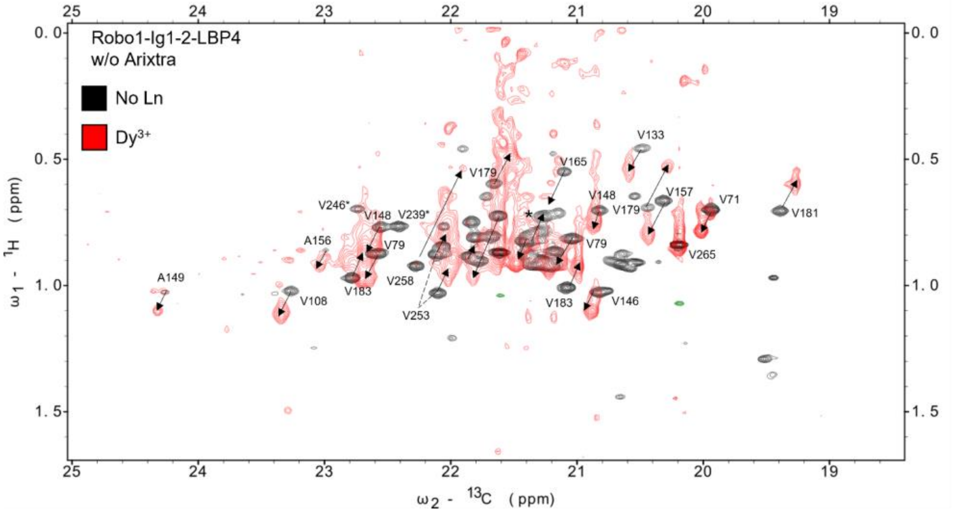

Figure S1. Overlay of HETCOR spectra of hRobo1-Ig1-2-LBP4 without Arixtra showing PCS. Peaks without lanthanide are indicated by black contours while peaks with Dy^{3+} are drawn in red. The black arrows connect diamagnetic and paramagnetic peaks for the same resonance. Several signals disappear in the Dy^{3+} spectrum and are indicated by asterisks.

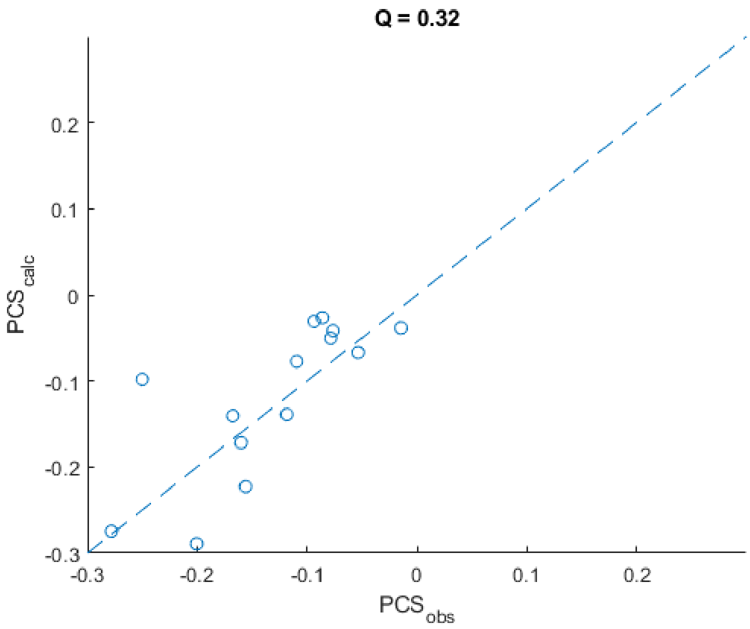

Figure S2. Correlation plot showing agreement between observed and predicted Ig2 PCS values (combined Arixtra-bound and unbound measurements).

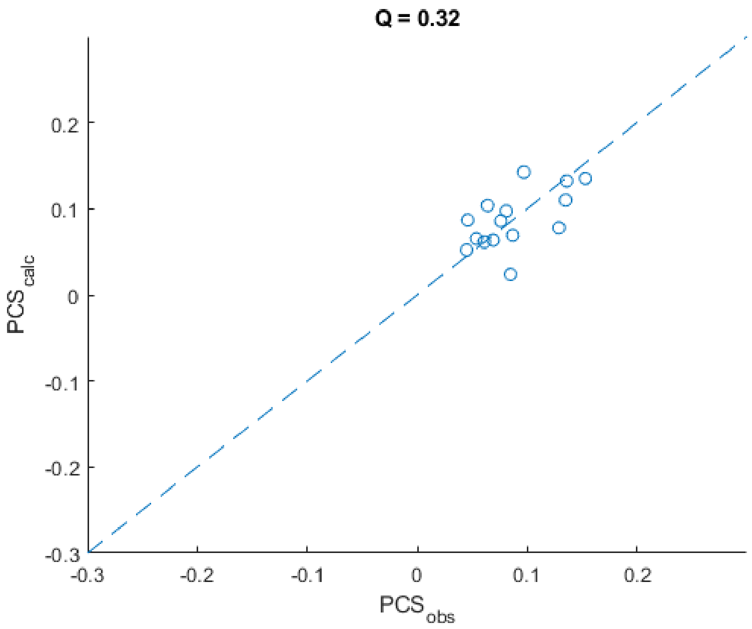

Figure S3. PCS correlation plot showing agreement for Ig1 PCS values in the unbound state determined from the best-fitting model.

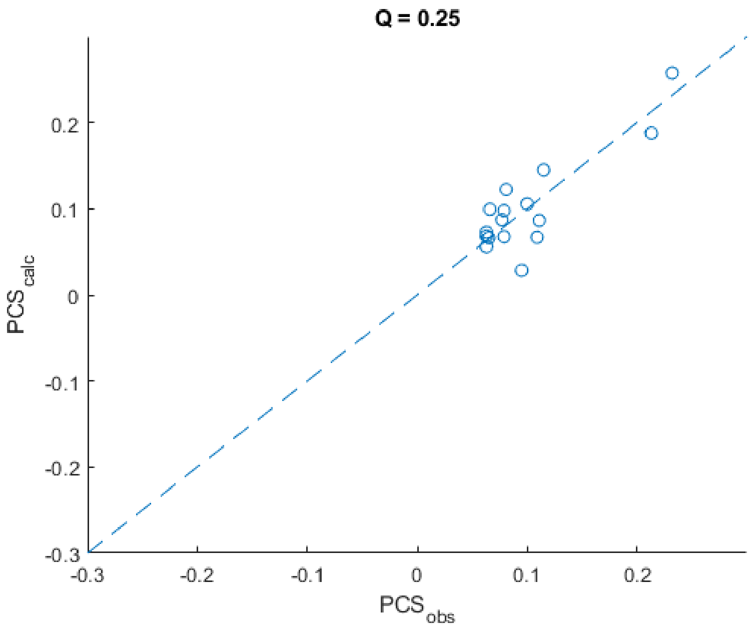

Figure S4. PCS correlation plot showing agreement for Ig1 PCS values in the Arixtra-bound state determined from the best-fitting model.

Table S1. Data used for resonance assignment. Peaks are labeled with their final assignments. While the numbers 1 and 2 for valines indicate γ1 and γ2 methyls, in most cases correlations are not sufficiently strong to make stereospecific assignments. Correlations that were not observed are indicated with N/A.

Residues	$^{13}C_\gamma$ Shift (ppm)		$^{1}H_\gamma$ Shift (ppm)		$^{1}H_\beta$ Shift (ppm)		$^{1}H_\alpha$ Shift (ppm)	
	**Expt.**	**Pred.**	**Expt.**	**Pred.**	**Expt.**	**Pred.**	**Expt.**	**Pred.**
V71-1	18.74	21.13	0.90	0.82	2.04	2.02	4.32	4.13
V71-2	17.30	21.16	0.77	0.80	2.03	2.02	4.32	4.13
V79-1	18.26	20.85	0.84	0.68	2.04	1.91	N/A	4.50
V79-2	19.80	21.33	0.96	0.67	2.05	1.91	4.46	4.50
V108-1	18.78	21.78	0.89	0.87	1.96	2.03	3.93	4.16
V108-2	20.58	21.82	1.10	0.91	1.96	2.03	3.91	4.16
V133-1	18.66	21.69	0.81	0.85	1.94	2.01	4.01	4.08
V133-2	17.84	21.55	0.57	0.56	1.95	2.01	4.02	4.08
V144-1	18.51	22.01	1.00	0.97	1.98	1.98	4.88	4.79
V144-2	19.13	21.75	0.91	0.83	1.98	1.98	4.88	4.79
V148-1	19.89	21.39	0.85	0.85	1.96	1.86	4.86	4.71
V148-2	18.17	21.39	0.78	0.80	1.96	1.86	4.86	4.71
V146-1	18.51	21.59	0.93	0.73	1.96	2.03	4.70	4.91
V146-2	18.21	21.42	1.10	0.59	1.96	2.03	4.70	4.91
V157-1	19.38	21.74	0.86	0.77	2.14	2.02	4.67	4.67
V157-2	17.93	20.27	0.74	0.68	2.14	2.02	4.66	4.67
V165-1	19.07	21.67	0.57	0.60	1.51	1.91	4.61	4.88
V165-2	18.53	21.32	0.59	0.71	1.49	1.91	4.61	4.88
V179-1	20.46	21.64	0.72	0.73	1.84	1.95	N/A	4.38
V179-2	21.62	21.85	0.63	0.62	1.84	1.95	N/A	4.38
V181-1	21.73	21.18	0.93	0.85	2.18	2.06	N/A	4.42
V181-2	19.30	20.29	0.74	0.70	2.18	2.06	N/A	4.42
V183-1	22.71	21.75	1.00	0.88	1.81	1.97	N/A	3.82
V183-2	21.02	21.52	1.04	0.92	1.81	1.97	N/A	3.82
V188-1	21.12	21.54	0.82	0.77	1.77	1.86	N/A	4.81
V188-2	21.63	21.50	0.84	0.69	1.77	1.86	N/A	4.81
V239-1	22.15	21.64	0.77	0.75	1.67	1.74	N/A	4.47
V239-2	21.93	21.40	0.80	0.70	1.67	1.74	N/A	4.47
V241-1	21.42	21.61	0.69	0.83	1.91	1.92	N/A	4.93
V241-2	21.06	21.57	0.75	0.72	1.91	1.92	N/A	4.93
V246-1	22.76	22.0	0.72	0.93	2.15	2.24	N/A	3.81
V246-2	21.87	21.56	0.92	0.87	2.15	2.24	N/A	3.81

V253-1	22.10	21.31	1.06	0.91	1.73	1.91	N/A	4.25
V253-2	22.14	21.53	0.91	0.86	1.73	1.91	N/A	4.25
V258-1	22.23	21.42	0.96	0.84	1.84	1.88	N/A	5.08
V258-2	21.97	21.07	0.88	0.69	1.84	1.88	N/A	5.08
V265-1	21.54	N/A	0.90	N/A	1.98	N/A	N/A	N/A
V265-2	20.13	N/A	0.87	N/A	1.98	N/A	N/A	N/A

Table S2. Chemical shifts for hRobo1-Ig1-2-LBP4.

		**Chemical Shifts**			
**Residue**	**Nuclei**	**ω_1 (^{1}H) (ppm)**	**ω_2 (^{13}C) (ppm)**	**^{1}H Line Width (ppm)**	**^{13}C Line Width (ppm)**
V71	$H_{\gamma1}$-$C_{\gamma1}$	0.86	21.41	0.02	0.07
	$H_{\gamma2}$-$C_{\gamma2}$	0.74	19.94	0.02	0.06
V79	$H_{\gamma1}$-$C_{\gamma1}$	0.85	21.04	0.02	0.08
	$H_{\gamma2}$-$C_{\gamma2}$	0.91	22.6	0.02	0.1
A85	H_{β}-C_{β}	1.07	23.6	0.03	0.1
A91	H_{β}-C_{β}	1.24	21.99	0.03	0.09
V108	$H_{\gamma1}$-$C_{\gamma1}$	1.06	23.27	0.03	0.09
	$H_{\gamma2}$-$C_{\gamma2}$	0.92	21.40	0.02	0.06
V133	$H_{\gamma1}$-$C_{\gamma1}$	0.76	21.26	0.02	0.08
	$H_{\gamma2}$-$C_{\gamma2}$	0.49	20.5	0.03	0.1
V144	$H_{\gamma1}$-$C_{\gamma1}$	0.78	21.84	0.02	0.06
	$H_{\gamma2}$-$C_{\gamma2}$	0.84	21.68	0.03	0.09
V146	$H_{\gamma1}$-$C_{\gamma1}$	0.90	21.17	0.02	0.06
	$H_{\gamma2}$-$C_{\gamma2}$	1.06	20.8	0.02	0.1
V148	$H_{\gamma1}$-$C_{\gamma1}$	0.74	20.82	0.02	0.08
	$H_{\gamma2}$-$C_{\gamma2}$	0.81	22.56	0.03	0.07
A149	H_{β}-C_{β}	1.06	24.3	0.03	0.1
A156	H_{β}-C_{β}	0.90	23.0	0.03	0.1
V157	$H_{\gamma1}$-$C_{\gamma1}$	0.70	20.32	0.02	0.07
	$H_{\gamma2}$-$C_{\gamma2}$	0.95	21.3	0.03	0.1
A161	H_{β}-C_{β}	1.28	23.1	0.03	0.1
V165	$H_{\gamma1}$-$C_{\gamma1}$	0.49	21.9	0.03	0.1
	$H_{\gamma2}$-$C_{\gamma2}$	0.59	21.10	0.03	0.09
A166	H_{β}-C_{β}	1.48	20.66	0.02	0.09
V179	$H_{\gamma1}$-$C_{\gamma1}$	0.73	20.4	0.03	0.1
	$H_{\gamma2}$-$C_{\gamma2}$	0.63	21.66	0.03	0.09

V181	$H_{\gamma1}$-$C_{\gamma1}$	0.74	19.39	0.02	0.08
	$H_{\gamma2}$-$C_{\gamma2}$	0.94	21.78	0.02	0.08
A182	H_{β}-C_{β}	1.26	20.1	0.03	0.1
V183	$H_{\gamma1}$-$C_{\gamma1}$	1.04	21.08	0.02	0.06
	$H_{\gamma2}$-$C_{\gamma2}$	1.01	22.78	0.02	0.07
A187	H_{β}-C_{β}	1.21	22.53	*N/A*	*N/A*
V188	$H_{\gamma1}$-$C_{\gamma1}$	0.85	21.80	0.02	0.06
	$H_{\gamma2}$-$C_{\gamma2}$	0.82	21.3	0.03	0.1
A Loop 1	H_{β}-C_{β}	1.33	19.52	0.02	0.09
A Loop 2	H_{β}-C_{β}	1.39	19.5	0.06	0.1
A235	H_{β}-C_{β}	1.07	23.4	0.03	0.1
V239	$H_{\gamma1}$-$C_{\gamma1}$	0.80	22.41	0.02	0.07
	$H_{\gamma2}$-$C_{\gamma2}$	0.80	22.06	0.03	0.08
V241	$H_{\gamma1}$-$C_{\gamma1}$	0.76	21.62	0.03	0.08
	$H_{\gamma2}$-$C_{\gamma2}$	0.75	21.15	0.03	0.09
V246	$H_{\gamma1}$-$C_{\gamma1}$	0.73	22.7	0.03	0.1
	$H_{\gamma2}$-$C_{\gamma2}$	0.92	21.85	0.02	0.07
V253	$H_{\gamma1}$-$C_{\gamma1}$	0.96	22.28	0.02	0.07
	$H_{\gamma2}$-$C_{\gamma2}$	0.88	22.07	0.03	0.07
A254	H_{β}-C_{β}	1.65	19.77	*N/A*	*N/A*
V258	$H_{\gamma1}$-$C_{\gamma1}$	0.91	22.11	0.02	0.07
	$H_{\gamma2}$-$C_{\gamma2}$	1.06	22.10	0.02	0.07
V265	$H_{\gamma1}$-$C_{\gamma1}$	0.90	21.61	0.01	0.06
	$H_{\gamma2}$-$C_{\gamma2}$	0.87	20.20	0.01	0.05

Table S3. Chemical shifts for hRobo1-Ig1-2-LBP4 with Dy^{3+} .

		Chemical Shifts			
**Residue**	**Nuclei**	**ω_1 (^{1}H) (ppm)**	**ω_2 (^{13}C) (ppm)**	**^{1}H Line Width (ppm)**	**^{13}C Line Width (ppm)**
V71	$H_{\gamma1}$-$C_{\gamma1}$	0.94	21.48	0.03	0.05
	$H_{\gamma2}$-$C_{\gamma2}$	0.81	20.02	0.04	0.07
V79	$H_{\gamma1}$-$C_{\gamma1}$	0.94	21.14	0.04	0.09
	$H_{\gamma2}$-$C_{\gamma2}$	1.00	22.70	0.03	0.08
A85	H_β-C_β	1.21	23.77	0.05	0.08
A91	H_β-C_β	*N/A*	*N/A*	*N/A*	*N/A*
V108	$H_{\gamma1}$-$C_{\gamma1}$	1.14	23.34	0.07	0.09
	$H_{\gamma2}$-$C_{\gamma2}$	*N/A*	*N/A*	*N/A*	*N/A*
V133	$H_{\gamma1}$-$C_{\gamma1}$	*N/A*	*N/A*	*N/A*	*N/A*
	$H_{\gamma2}$-$C_{\gamma2}$	0.6	20.6	0.1	0.1
V144	$H_{\gamma1}$-$C_{\gamma1}$	0.92	21.98	0.06	0.09
	$H_{\gamma2}$-$C_{\gamma2}$	0.99	21.82	0.07	0.09
V146	$H_{\gamma1}$-$C_{\gamma1}$	0.95	21.22	0.05	0.08
	$H_{\gamma2}$-$C_{\gamma2}$	1.13	20.9	0.09	0.1
V148	$H_{\gamma1}$-$C_{\gamma1}$	0.78	20.87	0.05	0.08
	$H_{\gamma2}$-$C_{\gamma2}$	0.9	22.6	0.2	0.2
A149	H_β-C_β	1.13	24.32	0.04	0.07
A156	H_β-C_β	0.95	23.0	0.07	0.4
V157	$H_{\gamma1}$-$C_{\gamma1}$	0.8	20.44	0.1	0.08
	$H_{\gamma2}$-$C_{\gamma2}$	*N/A*	*N/A*	*N/A*	*N/A*
A161	H_β-C_β	1.53	23.29	0.04	0.09
V165	$H_{\gamma1}$-$C_{\gamma1}$	*N/A*	*N/A*	*N/A*	*N/A*
	$H_{\gamma2}$-$C_{\gamma2}$	*N/A*	*N/A*	*N/A*	*N/A*
A166	H_β-C_β	1.55	20.84	0.04	0.08
V179	$H_{\gamma1}$-$C_{\gamma1}$	0.56	20.3	0.05	0.1
	$H_{\gamma2}$-$C_{\gamma2}$	0.51	21.53	0.07	0.09

V181	$H_{\gamma1}$-$C_{\gamma1}$	0.63	19.3	0.09	0.1
	$H_{\gamma2}$-$C_{\gamma2}$	0.87	21.7	0.09	0.1
A182	H_{β}-C_{β}	1.24	20.12	*N/A*	*N/A*
V183	$H_{\gamma1}$-$C_{\gamma1}$	0.95	20.99	0.05	0.06
	$H_{\gamma2}$-$C_{\gamma2}$	0.91	22.68	0.04	0.06
A187	H_{β}-C_{β}	*N/A*	*N/A*	*N/A*	*N/A*
V188	$H_{\gamma1}$-$C_{\gamma1}$	*N/A*	*N/A*	*N/A*	*N/A*
	$H_{\gamma2}$-$C_{\gamma2}$	*N/A*	*N/A*	*N/A*	*N/A*
A Loop 1	H_{β}-C_{β}	*N/A*	*N/A*	*N/A*	*N/A*
A Loop 2	H_{β}-C_{β}	*N/A*	*N/A*	*N/A*	*N/A*
A235	H_{β}-C_{β}	1.03	23.36	0.04	0.09
V239	$H_{\gamma1}$-$C_{\gamma1}$	1.00	22.62	N/A	N/A
	$H_{\gamma2}$-$C_{\gamma2}$	*N/A*	*N/A*	*N/A*	*N/A*
V241	$H_{\gamma1}$-$C_{\gamma1}$	0.93	21.8	0.05	0.6
	$H_{\gamma2}$-$C_{\gamma2}$	*N/A*	*N/A*	*N/A*	*N/A*
V246	$H_{\gamma1}$-$C_{\gamma1}$	*N/A*	*N/A*	*N/A*	*N/A*
	$H_{\gamma2}$-$C_{\gamma2}$	*N/A*	*N/A*	*N/A*	*N/A*
V253	$H_{\gamma1}$-$C_{\gamma1}$	0.67	22.0	0.06	0.1
	$H_{\gamma2}$-$C_{\gamma2}$	*N/A*	*N/A*	*N/A*	*N/A*
A254	H_{β}-C_{β}	1.43	19.56	*N/A*	*N/A*
V258	$H_{\gamma1}$-$C_{\gamma1}$	0.83	22.05	0.08	0.09
	$H_{\gamma2}$-$C_{\gamma2}$	0.97	22.0	0.09	0.1
V265	$H_{\gamma1}$-$C_{\gamma1}$	0.91	21.60	0.05	0.06
	$H_{\gamma2}$-$C_{\gamma2}$	0.88	20.19	0.06	0.06

Table S4. Pseudocontact shifts for hRobo1-Ig1-2-LBP4.

		PCS			
**Residue**	**Nuclei**	**^{1}H (ppm)**	**^{13}C (ppm)**	**^{1}H Error (ppm)** [a]	**^{13}C Error (ppm)**[a]
V71	$H_{\gamma1}$-$C_{\gamma1}$	0.08	0.1	0.05	0.1
	$H_{\gamma2}$-$C_{\gamma2}$	0.08	0.1	0.06	0.1
V79	$H_{\gamma1}$-$C_{\gamma1}$	0.09	0.1	0.06	0.2
	$H_{\gamma2}$-$C_{\gamma2}$	0.10	0.1	0.05	0.2
A85	H_{β}-C_{β}	0.14	0.1	0.07	0.2
A91	H_{β}-C_{β}	*N/A*	*N/A*	*N/A*	*N/A*
V108	$H_{\gamma1}$-$C_{\gamma1}$	0.09	0.1	0.09	0.2
	$H_{\gamma2}$-$C_{\gamma2}$	*N/A*	*N/A*	*N/A*	*N/A*
V133	$H_{\gamma1}$-$C_{\gamma1}$	*N/A*	*N/A*	*N/A*	*N/A*
	$H_{\gamma2}$-$C_{\gamma2}$	0.1	0.1	0.1	0.2
V144	$H_{\gamma1}$-$C_{\gamma1}$	0.14	0.1	0.08	0.2
	$H_{\gamma2}$-$C_{\gamma2}$	0.2	0.1	0.1	0.2
V146	$H_{\gamma1}$-$C_{\gamma1}$	0.05	0.0	0.07	0.1
	$H_{\gamma2}$-$C_{\gamma2}$	0.1	0.1	0.1	0.2
V148	$H_{\gamma1}$-$C_{\gamma1}$	0.05	0.0	0.07	0.2
	$H_{\gamma2}$-$C_{\gamma2}$	0.1	0.0	0.2	0.3
A149	H_{β}-C_{β}	0.07	0.1	0.06	0.2
A156	H_{β}-C_{β}	0.1	0.0	0.1	0.4
V157	$H_{\gamma1}$-$C_{\gamma1}$	0.1	0.1	0.1	0.2
	$H_{\gamma2}$-$C_{\gamma2}$	*N/A*	*N/A*	*N/A*	*N/A*
A161	H_{β}-C_{β}	0.24	0.2	0.07	0.2
V165	$H_{\gamma1}$-$C_{\gamma1}$	*N/A*	*N/A*	*N/A*	*N/A*
	$H_{\gamma2}$-$C_{\gamma2}$	*N/A*	*N/A*	*N/A*	*N/A*
A166	H_{β}-C_{β}	0.08	0.2	0.06	0.2
V179	$H_{\gamma1}$-$C_{\gamma1}$	-0.17	-0.2	0.09	0.2
	$H_{\gamma2}$-$C_{\gamma2}$	-0.1	-0.1	0.1	0.2

V181	$H_{\gamma1}$-$C_{\gamma1}$	-0.1	-0.1	0.1	0.2
	$H_{\gamma2}$-$C_{\gamma2}$	-0.1	-0.1	0.1	0.2
A182	H_{β}-C_{β}	-0.03	-0.02	*N/A*	*N/A*
V183	$H_{\gamma1}$-$C_{\gamma1}$	-0.09	-0.1	0.07	0.1
	$H_{\gamma2}$-$C_{\gamma2}$	-0.10	-0.1	0.07	0.1
A187	H_{β}-C_{β}	*N/A*	*N/A*	*N/A*	*N/A*
V188	$H_{\gamma1}$-$C_{\gamma1}$	*N/A*	*N/A*	*N/A*	*N/A*
	$H_{\gamma2}$-$C_{\gamma2}$	*N/A*	*N/A*	*N/A*	*N/A*
A Loop 1	H_{β}-C_{β}	*N/A*	*N/A*	*N/A*	*N/A*
A Loop 2	H_{β}-C_{β}	*N/A*	*N/A*	*N/A*	*N/A*
A235	H_{β}-C_{β}	-0.04	0.0	0.08	0.2
V239	$H_{\gamma1}$-$C_{\gamma1}$	0.20	0.2	0.02	0.1
	$H_{\gamma2}$-$C_{\gamma2}$	*N/A*	*N/A*	*N/A*	*N/A*
V241	$H_{\gamma1}$-$C_{\gamma1}$	0.17	0.2	0.08	0.7
	$H_{\gamma2}$-$C_{\gamma2}$	*N/A*	*N/A*	*N/A*	*N/A*
V246	$H_{\gamma1}$-$C_{\gamma1}$	*N/A*	*N/A*	*N/A*	*N/A*
	$H_{\gamma2}$-$C_{\gamma2}$	*N/A*	*N/A*	*N/A*	*N/A*
V253	$H_{\gamma1}$-$C_{\gamma1}$	-0.28	-0.2	0.08	0.2
	$H_{\gamma2}$-$C_{\gamma2}$	*N/A*	*N/A*	*N/A*	*N/A*
A254	H_{β}-C_{β}	-0.22	-0.21	*N/A*	*N/A*
V258	$H_{\gamma1}$-$C_{\gamma1}$	-0.1	-0.1	0.1	0.2
	$H_{\gamma2}$-$C_{\gamma2}$	-0.1	-0.1	0.1	0.2
V265	$H_{\gamma1}$-$C_{\gamma1}$	0.01	0.0	0.06	0.1
	$H_{\gamma2}$-$C_{\gamma2}$	0.00	0.0	0.07	0.1

[a] Errors were calculated as the sum of linewidths for the diamagnetic and paramagnetic peaks.

Table S5. Chemical Shifts for hRobo1-Ig1-2-LBP4, Arixtra bound form.

		Chemical Shifts			
**Residue**	**Nuclei**	**ω_1 (^{1}H) (ppm)**	**ω_2 (^{13}C) (ppm)**	**^{1}H Line Width (ppm)**	**^{13}C Line Width (ppm)**
V71	$H_{\gamma 1}$-$C_{\gamma 1}$	0.86	21.41	0.03	0.05
	$H_{\gamma 2}$-$C_{\gamma 2}$	0.73	19.95	0.03	0.05
V79	$H_{\gamma 1}$-$C_{\gamma 1}$	0.85	21.02	0.02	0.06
	$H_{\gamma 2}$-$C_{\gamma 2}$	0.91	22.6	0.03	0.1
A85	H_{β}-C_{β}	1.06	23.72	0.03	0.09
A91	H_{β}-C_{β}	1.24	21.99	0.03	0.07
V108	$H_{\gamma 1}$-$C_{\gamma 1}$	1.06	23.34	0.05	0.07
	$H_{\gamma 2}$-$C_{\gamma 2}$	0.9	21.39	0.1	0.02
V133	$H_{\gamma 1}$-$C_{\gamma 1}$	0.76	21.2	0.05	0.2
	$H_{\gamma 2}$-$C_{\gamma 2}$	0.46	20.5	0.04	0.1
V144	$H_{\gamma 1}$-$C_{\gamma 1}$	0.8	21.82	0.1	0.08
	$H_{\gamma 2}$-$C_{\gamma 2}$	0.83	21.64	0.02	0.09
V146	$H_{\gamma 1}$-$C_{\gamma 1}$	0.90	21.19	0.02	0.06
	$H_{\gamma 2}$-$C_{\gamma 2}$	1.06	20.83	0.03	0.06
V148	$H_{\gamma 1}$-$C_{\gamma 1}$	0.74	20.83	0.03	0.07
	$H_{\gamma 2}$-$C_{\gamma 2}$	0.81	22.57	0.05	0.09
A149	H_{β}-C_{β}	1.06	24.26	0.03	0.08
A156	H_{β}-C_{β}	0.89	23.0	0.05	0.1
V157	$H_{\gamma 1}$-$C_{\gamma 1}$	0.72	20.4	0.03	0.1
	$H_{\gamma 2}$-$C_{\gamma 2}$	0.94	21.3	0.03	0.1
A161	H_{β}-C_{β}	1.28	23.1	0.03	0.1
V165	$H_{\gamma 1}$-$C_{\gamma 1}$	0.5	21.9	0.2	0.9
	$H_{\gamma 2}$-$C_{\gamma 2}$	0.53	21.07	0.09	0.07
A166	H_{β}-C_{β}	1.47	20.44	0.03	0.08
V179	$H_{\gamma 1}$-$C_{\gamma 1}$	0.72	20.41	0.03	0.09

	$H_{\gamma2}$-$C_{\gamma2}$	0.63	21.66	0.06	0.09
V181	$H_{\gamma1}$-$C_{\gamma1}$	0.74	19.40	0.03	0.05
	$H_{\gamma2}$-$C_{\gamma2}$	0.94	21.78	0.03	0.06
A182	H_{β}-C_{β}	1.25	20.14	0.03	0.06
V183	$H_{\gamma1}$-$C_{\gamma1}$	1.04	21.08	0.02	0.05
	$H_{\gamma2}$-$C_{\gamma2}$	1.00	22.78	0.02	0.05
A187	H_{β}-C_{β}	1.1	23.4	0.2	0.2
V188	$H_{\gamma1}$-$C_{\gamma1}$	0.77	21.68	0.03	0.07
	$H_{\gamma2}$-$C_{\gamma2}$	0.82	21.31	0.03	0.09
A Loop 1	H_{β}-C_{β}	1.33	19.53	0.02	0.06
A Loop 2	H_{β}-C_{β}	1.40	19.5	0.03	0.2
A235	H_{β}-C_{β}	1.21	22.54	0.03	0.09
V239	$H_{\gamma1}$-$C_{\gamma1}$	0.80	22.39	0.03	0.06
	$H_{\gamma2}$-$C_{\gamma2}$	0.8	22.1	0.1	0.1
V241	$H_{\gamma1}$-$C_{\gamma1}$	0.84	21.81	0.04	0.06
	$H_{\gamma2}$-$C_{\gamma2}$	0.74	21.1	0.04	0.1
V246	$H_{\gamma1}$-$C_{\gamma1}$	0.73	22.78	0.03	0.09
	$H_{\gamma2}$-$C_{\gamma2}$	0.92	21.86	0.02	0.05
V253	$H_{\gamma1}$-$C_{\gamma1}$	0.91	22.13	0.03	0.05
	$H_{\gamma2}$-$C_{\gamma2}$	1.06	22.12	0.02	0.05
A254	H_{β}-C_{β}	1.65	19.77	0.02	0.07
V258	$H_{\gamma1}$-$C_{\gamma1}$	0.95	22.28	0.03	0.06
	$H_{\gamma2}$-$C_{\gamma2}$	0.88	22.08	0.02	0.06
V265	$H_{\gamma1}$-$C_{\gamma1}$	0.90	21.61	0.02	0.05
	$H_{\gamma2}$-$C_{\gamma2}$	0.87	20.20	0.01	0.02

Table S6. Chemical shifts for hRobo1-Ig1-2-LBP4, Arixtra bound form, with Dy^{3+}.

		**Chemical Shifts**			
**Residue**	**Nuclei**	**ω_1 (^1H) (ppm)**	**ω_2 (^{13}C) (ppm)**	**^1H Line Width (ppm)**	**^{13}C Line Width (ppm)**
V71	$H_{\gamma1}$-$C_{\gamma1}$	0.94	21.5	0.03	0.1
	$H_{\gamma2}$-$C_{\gamma2}$	0.81	20.01	0.03	0.06
V79	$H_{\gamma1}$-$C_{\gamma1}$	0.95	21.1	0.04	0.09
	$H_{\gamma2}$-$C_{\gamma2}$	1.02	22.7	0.05	0.1
A85	H_{β}-C_{β}	1.16	23.9	0.04	0.1
A91	H_{β}-C_{β}	1.31	22.1	0.05	0.1
V108	$H_{\gamma1}$-$C_{\gamma1}$	1.15	23.43	0.05	0.09
	$H_{\gamma2}$-$C_{\gamma2}$	*N/A*	*N/A*	*N/A*	*N/A*
V133	$H_{\gamma1}$-$C_{\gamma1}$	0.87	21.3	0.05	0.2
	$H_{\gamma2}$-$C_{\gamma2}$	0.56	20.6	0.09	0.1
V144	$H_{\gamma1}$-$C_{\gamma1}$	*N/A*	*N/A*	*N/A*	*N/A*
	$H_{\gamma2}$-$C_{\gamma2}$	1.04	21.86	0.07	0.13
V146	$H_{\gamma1}$-$C_{\gamma1}$	0.96	21.25	0.06	0.10
	$H_{\gamma2}$-$C_{\gamma2}$	1.14	20.89	0.04	0.09
V148	$H_{\gamma1}$-$C_{\gamma1}$	0.80	20.89	0.06	0.09
	$H_{\gamma2}$-$C_{\gamma2}$	0.88	22.62	0.05	0.08
A149	H_{β}-C_{β}	1.12	24.3	0.04	0.1
A156	H_{β}-C_{β}	0.95	23.0	0.04	0.1
V157	$H_{\gamma1}$-$C_{\gamma1}$	0.83	20.51	0.05	0.08
	$H_{\gamma2}$-$C_{\gamma2}$	*N/A*	*N/A*	*N/A*	*N/A*
A161	H_{β}-C_{β}	1.50	23.27	*N/A*	*N/A*
V165	$H_{\gamma1}$-$C_{\gamma1}$	*N/A*	*N/A*	*N/A*	*N/A*
	$H_{\gamma2}$-$C_{\gamma2}$	0.65	21.2	0.2	0.2
A166	H_{β}-C_{β}	*N/A*	*N/A*	*N/A*	*N/A*
V179	$H_{\gamma1}$-$C_{\gamma1}$	0.55	20.3	0.06	0.1

	$H_{\gamma2}$-$C_{\gamma2}$	0.51	21.54	0.04	0.08
V181	$H_{\gamma1}$-$C_{\gamma1}$	0.63	19.27	0.05	0.08
	$H_{\gamma2}$-$C_{\gamma2}$	0.89	21.74	0.05	0.07
A182	H_{β}-C_{β}	*N/A*	*N/A*	*N/A*	*N/A*
V183	$H_{\gamma1}$-$C_{\gamma1}$	0.96	21	0.05	0.07
	$H_{\gamma2}$-$C_{\gamma2}$	0.91	22.68	0.05	0.08
A187	H_{β}-C_{β}	0.82	23.23	*N/A*	*N/A*
V188	$H_{\gamma1}$-$C_{\gamma1}$	0.61	21.5	0.09	0.1
	$H_{\gamma2}$-$C_{\gamma2}$	*N/A*	*N/A*	*N/A*	*N/A*
A Loop 1	H_{β}-C_{β}	*N/A*	*N/A*	*N/A*	*N/A*
A Loop 2	H_{β}-C_{β}	*N/A*	*N/A*	*N/A*	*N/A*
A235	H_{β}-C_{β}	*N/A*	*N/A*	*N/A*	*N/A*
V239	$H_{\gamma1}$-$C_{\gamma1}$	1.1	22.7	0.1	0.1
	$H_{\gamma2}$-$C_{\gamma2}$	*N/A*	*N/A*	*N/A*	*N/A*
V241	$H_{\gamma1}$-$C_{\gamma1}$	0.7	21.7	0.2	0.2
	$H_{\gamma2}$-$C_{\gamma2}$	1.1	21.5	0.1	0.1
V246	$H_{\gamma1}$-$C_{\gamma1}$	*N/A*	*N/A*	*N/A*	*N/A*
	$H_{\gamma2}$-$C_{\gamma2}$	*N/A*	*N/A*	*N/A*	*N/A*
V253	$H_{\gamma1}$-$C_{\gamma1}$	0.83	22.1	0.08	0.1
	$H_{\gamma2}$-$C_{\gamma2}$	1.00	22.1	0.07	0.2
A254	H_{β}-C_{β}	1.46	19.5	0.05	0.1
V258	$H_{\gamma1}$-$C_{\gamma1}$	0.63	21.95	0.05	0.09
	$H_{\gamma2}$-$C_{\gamma2}$	0.60	21.81	0.06	0.08
V265	$H_{\gamma1}$-$C_{\gamma1}$	0.91	21.61	0.02	0.06
	$H_{\gamma2}$-$C_{\gamma2}$	0.88	20.19	0.03	0.05

Table S7. Pseudocontact shifts for hRobo1-Ig1-2-LBP4, Arixtra bound form.

		PCS			
**Residue**	**Nuclei**	**^{1}H (ppm)**	**^{13}C (ppm)**	**^{1}H Error (ppm) [a]**	**^{13}C Error (ppm) [a]**
V71	$H_{\gamma1}$-$C_{\gamma1}$	0.08	0.1	0.06	0.1
	$H_{\gamma2}$-$C_{\gamma2}$	0.08	0.1	0.06	0.1
V79	$H_{\gamma1}$-$C_{\gamma1}$	0.11	0.1	0.06	0.1
	$H_{\gamma2}$-$C_{\gamma2}$	0.12	0.1	0.08	0.2
A85	H_{β}-C_{β}	0.10	0.1	0.07	0.2
A91	H_{β}-C_{β}	0.06	0.1	0.07	0.2
V108	$H_{\gamma1}$-$C_{\gamma1}$	0.10	0.1	0.09	0.2
	$H_{\gamma2}$-$C_{\gamma2}$	*N/A*	*N/A*	*N/A*	*N/A*
V133	$H_{\gamma1}$-$C_{\gamma1}$	0.1	0.1	0.1	0.4
	$H_{\gamma2}$-$C_{\gamma2}$	0.09	0.1	0.1	0.3
V144	$H_{\gamma1}$-$C_{\gamma1}$	*N/A*	*N/A*	*N/A*	*N/A*
	$H_{\gamma2}$-$C_{\gamma2}$	0.2	0.2	0.1	0.2
V146	$H_{\gamma1}$-$C_{\gamma1}$	0.07	0.1	0.08	0.2
	$H_{\gamma2}$-$C_{\gamma2}$	0.08	0.1	0.07	0.1
V148	$H_{\gamma1}$-$C_{\gamma1}$	0.06	0.1	0.09	0.2
	$H_{\gamma2}$-$C_{\gamma2}$	0.1	0.1	0.1	0.2
A149	H_{β}-C_{β}	0.07	0.1	0.07	0.2
A156	H_{β}-C_{β}	0.06	0.0	0.08	0.2
V157	$H_{\gamma1}$-$C_{\gamma1}$	0.11	0.1	0.09	0.2
	$H_{\gamma2}$-$C_{\gamma2}$	*N/A*	*N/A*	*N/A*	*N/A*
A161	H_{β}-C_{β}	0.2	0.2	*N/A*	*N/A*
V165	$H_{\gamma1}$-$C_{\gamma1}$	*N/A*	*N/A*	*N/A*	*N/A*
	$H_{\gamma2}$-$C_{\gamma2}$	0.1	0.1	0.2	0.3
A166	H_{β}-C_{β}	*N/A*	*N/A*	*N/A*	*N/A*
V179	$H_{\gamma1}$-$C_{\gamma1}$	-0.17	-0.1	0.09	0.2
	$H_{\gamma2}$-$C_{\gamma2}$	-0.1	-0.1	0.1	0.2
V181	$H_{\gamma1}$-$C_{\gamma1}$	-0.11	-0.1	0.07	0.1

	$H_{\gamma 2}$-$C_{\gamma 2}$	-0.05	0.0	0.08	0.1
A182	H_{β}-C_{β}	*N/A*	*N/A*	N/A	N/A
V183	$H_{\gamma 1}$-$C_{\gamma 1}$	-0.08	-0.1	0.08	0.1
	$H_{\gamma 2}$-$C_{\gamma 2}$	-0.09	-0.1	0.07	0.1
A187	H_{β}-C_{β}	-0.3	-0.2	*N/A*	*N/A*
V188	$H_{\gamma 1}$-$C_{\gamma 1}$	-0.2	-0.2	0.1	0.2
	$H_{\gamma 2}$-$C_{\gamma 2}$	*N/A*	*N/A*	*N/A*	*N/A*
A Loop 1	H_{β}-C_{β}	*N/A*	*N/A*	*N/A*	*N/A*
A Loop 2	H_{β}-C_{β}	*N/A*	*N/A*	*N/A*	*N/A*
A235	H_{β}-C_{β}	*N/A*	*N/A*	*N/A*	*N/A*
V239	$H_{\gamma 1}$-$C_{\gamma 1}$	0.3	0.3	0.1	0.2
	$H_{\gamma 2}$-$C_{\gamma 2}$	*N/A*	*N/A*	*N/A*	*N/A*
V241	$H_{\gamma 1}$-$C_{\gamma 1}$	-0.2	-0.2	0.2	0.2
	$H_{\gamma 2}$-$C_{\gamma 2}$	0.3	0.4	0.1	0.2
V246	$H_{\gamma 1}$-$C_{\gamma 1}$	*N/A*	*N/A*	*N/A*	*N/A*
	$H_{\gamma 2}$-$C_{\gamma 2}$	*N/A*	*N/A*	*N/A*	*N/A*
V253	$H_{\gamma 1}$-$C_{\gamma 1}$	-0.1	-0.1	0.1	0.2
	$H_{\gamma 2}$-$C_{\gamma 2}$	-0.07	-0.1	0.09	0.3
A254	H_{β}-C_{β}	-0.18	-0.2	0.07	0.2
V258	$H_{\gamma 1}$-$C_{\gamma 1}$	-0.32	-0.3	0.09	0.1
	$H_{\gamma 2}$-$C_{\gamma 2}$	-0.28	-0.3	0.08	0.1
V265	$H_{\gamma 1}$-$C_{\gamma 1}$	0.01	0.0	0.04	0.1
	$H_{\gamma 2}$-$C_{\gamma 2}$	0.01	0.00	0.04	0.07

[a] Errors were calculated as the sum of linewidths for the diamagnetic and paramagnetic peaks.

Table S8. Ig2 PCS data used for magnetic susceptibility tensor solution.

**Residue**	**Nuclei**	**PCS**	
		**^{1}H (ppm)**	**^{13}C (ppm)**
A166	H_β-C_β	*N/A*	*N/A*
V179	$H_{\gamma1}$-$C_{\gamma1}$	-0.17	-0.1
	$H_{\gamma2}$-$C_{\gamma2}$	-0.12	-0.13
V181	$H_{\gamma1}$-$C_{\gamma1}$	-0.11	-0.12
	$H_{\gamma2}$-$C_{\gamma2}$	-0.05	-0.05
A182	H_β-C_β	-0.01	-0.01
V183	$H_{\gamma1}$-$C_{\gamma1}$	-0.09	-0.09
	$H_{\gamma2}$-$C_{\gamma2}$	-0.09	-0.10
A187	H_β-C_β	-0.25	-0.19
V188	$H_{\gamma1}$-$C_{\gamma1}$	-0.16	-0.15
	$H_{\gamma2}$-$C_{\gamma2}$	*N/A*	*N/A*
A Loop 1	H_β-C_β	*N/A*	*N/A*
A Loop 2	H_β-C_β	*N/A*	*N/A*
A235	H_β-C_β	*N/A*	*N/A*
V239	$H_{\gamma1}$-$C_{\gamma1}$	0.25	0.25
	$H_{\gamma2}$-$C_{\gamma2}$	*N/A*	*N/A*
V241	$H_{\gamma1}$-$C_{\gamma1}$	-0.16	-0.15
	$H_{\gamma2}$-$C_{\gamma2}$	0.33	0.35
V246	$H_{\gamma1}$-$C_{\gamma1}$	-0.34	-0.30
	$H_{\gamma2}$-$C_{\gamma2}$	*N/A*	*N/A*
V253	$H_{\gamma1}$-$C_{\gamma1}$	-0.08	-0.07
	$H_{\gamma2}$-$C_{\gamma2}$	-0.08	-0.08
A254	H_β-C_β	-0.20	-0.22
V258	$H_{\gamma1}$-$C_{\gamma1}$	-0.30	-0.29
	$H_{\gamma2}$-$C_{\gamma2}$	-0.28	-0.27
V265	$H_{\gamma1}$-$C_{\gamma1}$	0.01	0.00
	$H_{\gamma2}$-$C_{\gamma2}$	0.01	-0.01

Table S9. Q scores and Euler angles for top 20 Ig1 orientations found using unbound PCS data.

**Rank**	**Q**	**α (degrees)**	**β (degrees)**	**γ (degrees)**
1	0.32	-56.9	36.7	34.9
2	0.32	-56.9	25.7	38.6
3	0.32	-56.9	22.0	38.6
4	0.32	-60.6	25.7	42.2
5	0.32	-56.9	33.1	34.9
6	0.32	-60.6	36.7	38.6
7	0.32	-60.6	22.0	42.2
8	0.32	-56.9	40.4	34.9
9	0.32	-53.3	25.7	34.9
10	0.32	-53.3	22.0	34.9
11	0.32	-53.3	36.7	31.2
12	0.32	-60.6	33.1	38.6
13	0.33	-56.9	47.8	31.2
14	0.33	-56.9	29.4	38.6
15	0.33	-53.3	40.4	31.2
16	0.33	-56.9	51.4	31.2
17	0.33	-60.6	47.8	34.9
18	0.33	-53.3	33.1	31.2
19	0.33	-64.3	25.7	45.9
20	0.33	-64.3	22.0	45.9

Table S10. Q scores and Euler angles for top 20 Ig1 orientations found using Arixtra-bound PCS data.

Rank	Q	α (degrees)	β (degrees)	γ (degrees)
1	0.25	-75.3	18.4	56.9
2	0.25	-68.0	29.4	45.9
3	0.25	-71.6	18.4	53.3
4	0.25	-71.6	29.4	49.6
5	0.25	-79.0	18.4	60.6
6	0.25	-75.3	22.0	56.9
7	0.25	-71.6	22.0	53.3
8	0.25	-79.0	22.0	60.6
9	0.25	-68.0	18.4	49.6
10	0.25	-64.3	29.4	42.2
11	0.26	-68.0	25.7	45.9
12	0.26	-71.6	25.7	49.6
13	0.26	-82.7	18.4	64.3
14	0.26	-68.0	22.0	49.6
15	0.26	-75.3	29.4	53.3
16	0.26	-82.7	22.0	64.3
17	0.26	-68.0	36.7	42.2
18	0.26	-64.3	25.7	42.2
19	0.26	-75.3	25.7	53.3
20	0.26	-90.0	11.0	75.3
